# Rare variants in *SOX17* are associated with pulmonary arterial hypertension with congenital heart disease

**DOI:** 10.1186/s13073-018-0566-x

**Published:** 2018-07-20

**Authors:** Na Zhu, Carrie L. Welch, Jiayao Wang, Philip M. Allen, Claudia Gonzaga-Jauregui, Lijiang Ma, Alejandra K. King, Usha Krishnan, Erika B. Rosenzweig, D. Dunbar Ivy, Eric D. Austin, Rizwan Hamid, Michael W. Pauciulo, Katie A. Lutz, William C. Nichols, Jeffrey G. Reid, John D. Overton, Aris Baras, Frederick E. Dewey, Yufeng Shen, Wendy K. Chung

**Affiliations:** 1 Department of Pediatrics, Columbia University Medical Center, New York, NY USA; 2 Department of Systems Biology, Columbia University Medical Center, New York, NY USA; 3 Regeneron Genetics Center, Regeneron Pharmaceuticals, Tarrytown, New York, USA; 4 Department of Medicine, Columbia University Medical Center, New York, NY USA; 5 Department of Pediatric Cardiology, Children’s Hospital Colorado, Denver, CO USA; 6 Department of Pediatrics, Vanderbilt University School of Medicine, Nashville, TN USA; 7 Division of Human Genetics, Cincinnati Children’s Hospital Medical Center, Cincinnati, OH USA; 8 Department of Pediatrics, University of CincinnatiCollege of Medicine, Cincinnati, OH USA; 9 Department of Biomedical Informatics, Columbia University, New York, NY USA; 10 Herbert Irving Comprehensive Cancer Center, Columbia University Medical Center, New York, NY USA; 11 New York, USA

**Keywords:** Pulmonary hypertension, Congenital heart disease, Exome sequencing, Genetic association study

## Abstract

**Background:**

Pulmonary arterial hypertension (PAH) is a rare disease characterized by distinctive changes in pulmonary arterioles that lead to progressive pulmonary arterial pressures, right-sided heart failure, and a high mortality rate. Up to 30% of adult and 75% of pediatric PAH cases are associated with congenital heart disease (PAH-CHD), and the underlying etiology is largely unknown. There are no known major risk genes for PAH-CHD.

**Methods:**

To identify novel genetic causes of PAH-CHD, we performed whole exome sequencing in 256 PAH-CHD patients. We performed a case-control gene-based association test of rare deleterious variants using 7509 gnomAD whole genome sequencing population controls. We then screened a separate cohort of 413 idiopathic and familial PAH patients without CHD for rare deleterious variants in the top association gene.

**Results:**

We identified *SOX17* as a novel candidate risk gene (*p* = 5.5e−7). *SOX17* is highly constrained and encodes a transcription factor involved in Wnt/β-catenin and Notch signaling during development. We estimate that rare deleterious variants contribute to approximately 3.2% of PAH-CHD cases. The coding variants identified include likely gene-disrupting (LGD) and deleterious missense, with most of the missense variants occurring in a highly conserved HMG-box protein domain. We further observed an enrichment of rare deleterious variants in putative targets of SOX17, many of which are highly expressed in developing heart and pulmonary vasculature. In the cohort of PAH without CHD, rare deleterious variants of *SOX17* were observed in 0.7% of cases.

**Conclusions:**

These data strongly implicate *SOX17* as a new risk gene contributing to PAH-CHD as well as idiopathic/familial PAH. Replication in other PAH cohorts and further characterization of the clinical phenotype will be important to confirm the precise role of *SOX17* and better estimate the contribution of genes regulated by SOX17.

**Electronic supplementary material:**

The online version of this article (10.1186/s13073-018-0566-x) contains supplementary material, which is available to authorized users.

## Background

Pulmonary arterial hypertension (PAH[MIM:178600]) is a rare disease characterized by distinctive changes in pulmonary arterioles that lead to progressive pulmonary arterial pressures, right-sided heart failure and a high mortality rate. Up to 30% of adult- [1, 2] and 75% of pediatric-onset PAH cases [3] are associated with congenital heart disease (PAH-CHD), and due to improved treatments, the number of adults with PAH-CHD is rising [1, 4]. Congenital heart defects can result in left-to-right (systemic-to-pulmonary) shunts leading to increased pulmonary blood flow and risk of PAH. However, not all patients are exposed to prolonged periods of increased pulmonary flow. PAH may persist following surgical repair of cardiac defects or recur many years after repair. Thus, the underlying etiology is heterogeneous and may include increased pulmonary blood flow, pulmonary vasculature abnormalities, or a combination. In addition to environmental factors, genetic factors likely play an important role in PAH-CHD although no major risk gene has been identified to date [1].

Genetic studies of PAH alone have identified 11 known risk genes for PAH [5–8]. Several of the risk genes encode members of the transforming growth factor beta/bone morphogenetic protein (TGF-β/BMP) signaling pathway, important in both vasculogenesis and embryonic heart development. For example, mutations in bone morphogenetic protein receptor type 2 (*BMPR2*) are found in approximately 70% of familial and 10–40% of idiopathic PAH cases. Estimates of the frequency of *BMPR2* mutations in PAH-CHD are considerably lower than for PAH alone [9–11]. Mutations in other *TGFβ* family member genes—activin A, receptor type II-like 1 (*ACVRL1*), endoglin (*ENG*), BMP receptor type 1A (*BMPR1A*) and type 1B (*BMPR1B*)—as well as caveolin-1 (*CAV1*), eukaryotic initiation translation factor 2 alpha kinase 4 (*EIF2AK4*), potassium two-pore-domain channel subfamily K member 3 (*KCNK3*), SMAD family members 4 and 9 (*SMAD4* and *SMAD9*), and T-box4 (*TBX4*) have all been identified as less frequent or rare causes of PAH [5–8]. The genetics of CHD are complex and no single major risk gene accounts for more than 1% of cases [12, 13]. Aneuploidies and copy number variations underlie up to 23% of CHD cases [14, 15]. Rare, inherited, and de novo variants in hundreds of genes encoding transcription factors, chromatin regulators, signal transduction proteins, and cardiac structural proteins have been implicated in ~ 10% of CHD cases [12, 16–19].

To identify novel genetic causes of PAH-CHD, we performed exome sequencing in a patient cohort of PAH-CHD. Association analysis using population controls identified *SOX17*, a member of the SRY-related HMG-box family of transcription factors, as a new candidate risk gene.

## Methods

An overview of the experimental design and workflow is provided in Additional file 1: Figure S1.

### Patients

PAH-CHD patients were recruited from the pulmonary hypertension centers at Columbia University and Children’s Hospital of Colorado (via enrollment in the PAH Biobank at Cincinnati Children's Hospital Medical Center). Patients were diagnosed according to the World Health Organization (WHO) pulmonary hypertension group I classification [20]. The diagnosis of PAH-CHD was confirmed by medical record review including right heart catheterization and echocardiogram to define the cardiac anatomy. The cohort included 15 familial cases, 160 singletons with no family history of PAH, 61 trios (proband and two unaffected biological parents), and 20 duos (proband and one unaffected parent). Written informed consent (and assent when appropriate) was obtained under a protocol approved by the institutional review board at Columbia University Medical Center or Children’s Hospital of Colorado.

### Whole exome sequencing (WES)

Familial cases were screened for *BMPR2* and *ACVRL1* mutations by Sanger sequencing and multiplex ligation-dependent probe amplification (MLPA). Familial cases without mutations in the two risk genes and all other samples were exome sequenced. DNA was extracted from peripheral blood leukocytes using Puregene reagents (Gentra Systems Inc., Minnesota, USA). Exome sequencing was performed in collaboration with the Regeneron Genetics Center (RGC) or at the Children’s Hospital of Cincinnati. In brief, genomic DNA processed at the RGC was prepared with a customized reagent kit from Kapa Biosystems and captured using the SeqCap VCRome 2 exome capture reagent or xGen lockdown probes. Patient DNA samples sequenced at the PAH Biobank/Cincinnati Children’s Hospital Medical Center were prepared with the Clontech Advantage II kit and enriched using the SeqCap EZ exome V2 capture reagent. All samples were sequenced on the Illumina HiSeq 2500 platform, generating 76-bp paired-end reads. Read-depth coverage was ≥ 15× in ≥ 95% of targeted regions for all exome sequencing samples.

### WES data analysis

The workflow is outlined in Additional file 1: Figure S1A. We used a previously established bioinformatics procedure [18] to process and analyze exome sequence data. Specifically, we used BWA-MEM (Burrows-Wheeler Aligner) [21] to map and align paired-end reads to the human reference genome (version GRCh37/hg19), Picard MarkDuplicates to identify and flag PCR duplicate reads, GATK HaplotypeCaller (version 3) [22, 23] to call genetic variants, and GATK variant quality score recalibration (VQSR) to estimate accuracy of variant calls. We used heuristic filters to minimize potential technical artifacts, excluding variants that met any of the following conditions: missingness > 10%, minimum read depth ≤ 8 reads, allele balance ≤ 20% [24], genotype quality < 30, mappability < 1 (based on 150 bp fragments), or GATK VQSR < 99.6. Only variants with FILTER “PASS” in gnomAD WGS and restricted to the captured protein coding region were kept.

We used ANNOVAR [25] to annotate the variants and aggregate information about allele frequencies (AF) and in silico predictions of deleteriousness. We used population AF from public databases: Exome Aggregation Consortium (ExAC) [26] and Genome Aggregation Database (gnomAD). Rare variants were defined by AF < 0.01% in both ExAC and gnomAD WES datasets. We employed multiple in silico prediction algorithms including PolyPhen 2, metaSVM [27], Combined Annotation Dependent Depletion (CADD) [28], and REVEL (rare exome variant ensemble learner) [29]. We noted that REVEL outperformed other ensemble methods in pathogenicity prediction in a recent comparison using clinical genetic data [30]. We performed further evaluation of the prediction toolkits using de novo missense variants published in a recent CHD study [19] and published de novo variants of unaffected siblings of Simons Simplex Collection [31] as controls. We observed that REVEL-predicted damaging missense de novo variants reached the highest enrichment rate in cases compared to controls (Additional file 1: Figure S1B). Thus, we ultimately used REVEL to define damaging missense variants (D-mis, REVEL > 0.5) in this study.

We identified de novo variants in a set of 60 PAH-CHD trios using methods described previously [18, 32], and manually inspected all candidate de novo variants using the Integrative Genomics Viewer (IGV) [33] to exclude potential false positives.

### Identification of rare, deleterious variants in established risk genes

We screened for variants in 11 known risk genes for PAH [5–8]: *ACVRL1*, *BMPR1A*, *BMPR1B*, *BMPR2*, *CAV1*, *EIF2AK4*, *ENG*, *KCNK3*, *SMAD4*, *SMAD9*, and *TBX4*. We also screened for variants in the recently curated list of 253 candidate risk genes for CHD [19]. Variants identified in the PAH-CHD cohort were compared to mutations reported in the literature and in genetic databases (Online Mendelian Inheritance in Man database, Human Genome Mutation Database [34] and ClinVar [35]). We defined deleterious variants as likely gene-disrupting (LGD) (including premature stopgain, frameshift indels, canonical splicing variants, and deletion of exons) or damaging missense with REVEL score > 0.5 (D-mis). Insertion/deletion variants in known risk genes were confirmed with Sanger sequencing and tested for disease segregation when family DNA samples were available.

### Statistical analysis

To identify novel candidate risk genes, we performed a case-control association test comparing frequency of rare deleterious variants in each gene in PAH-CHD cases with gnomAD whole genome sequencing (WGS) subjects as population controls. To control for ethnicity, we selected cases of European ancestry (*n* = 144) using principal components analysis (PCA) (*Peddy* software package) [36] (Additional file 1: Figure S1C) and gnomAD subjects of non-Finnish European (NFE) ancestry (*n* = 7509). Since cases and controls were sequenced using different platforms, we assessed the batch effect based on the burden of rare synonymous variants, a variant class that is mostly neutral with respect to disease status. We observed that the frequency of rare synonymous variants in cases and controls was virtually identical (enrichment rate = 1.01, *p* value = 0.4) (Additional file 1: Table S3a). The analysis of disease-associated genes was confined to gene-specific enrichment of rare, deleterious variants (AF < 0.01%, LGD or D-mis). We assumed that under the null model, the number of rare deleterious variants observed in cases should follow a binomial distribution, given the total number of such variants in cases and controls, and a rate determined by fraction of cases in total number of subjects (cases and controls). The enrichment rate was then determined by the average number of variants in cases over the sum of average number of variants in cases and controls. The statistical significance of enrichment was tested using binom.test in R. We defined the threshold for genome-wide significance by Bonferroni correction for multiple testing (*n* = 17,701, threshold *p* value = 2.8e−6). We used the Benjamini-Hochberg procedure to estimate false discovery rate (FDR) by p.adjust in R. All *SOX17* variants reported herein were confirmed with Sanger sequencing and inheritance determined when parental DNA samples were available.

To guard against spurious association results due to population differences or batch effects inherent to the use of publicly available gnomAD data, we repeated the association analysis using a set of 1319 European control subjects with individual level data obtained from the same analytical pipeline and called jointly with the PAH-CHD cases. These controls were comprised of unrelated, unaffected European parents from the Pediatric Cardiac Genomics Consortium [18]. The data were captured using NimbleGen V2.0. We performed principle components analysis of ethnicity with cases and controls together.

To estimate the burden of de novo variants in cases, we calculated the background mutation rate using a previously published tri-nucleotide change table [32, 37] and calculated the rate in protein-coding regions that are uniquely mappable. We assumed that the number of de novo variants of various types (e.g., synonymous, missense, LGD) expected by chance in gene sets or all genes followed a Poisson distribution [32]. For a given type of de novo variant in a gene set, we set the observed number of cases to m1, the expected number to m0, estimated the enrichment rate by (m1/m0), and tested for significance using an exact Poisson test (poisson.test in R) with m0 as the expectation.

## Results

Characteristics of the PAH-CHD cohort are shown in Table 1. The cohort included 15 familial and 241 sporadic cases, including 61 parent-child trios and 20 duos. The majority of cases (56%) had an age of PAH onset < 18 years (pediatric-onset). There were more females among both pediatric-onset (*n* = 91/53, 1.7:1 female-to-male ratio) and adult-onset (*n* = 88/24, 3.7:1) patients, with a significant ~ 2-fold enrichment of females for adult- compared to pediatric-onset PAH (*p* = 0.009) (Table 1). Fifty-six percent of the patients were of European ancestry, 26% Hispanic, and 5–7% each of African, East Asian, or South Asian. The most common cardiac defects were atrial and ventricular septum defects; however, more severe defects were more frequent in pediatric-onset cases.

**Table 1:** PAH-CHD patient population

	Pediatric	Adult
Male, *n* (%)	53 (36.8)	24 (21.4)
Female, *n* (%)	91 (63.2)	88 (78.6)
Total, *n* (%)	144 (56.3)	112 (43.7)
Female-to-male ratio	1.7:1	3.7:1^a^
Ancestry, *n* (%)		
East Asian	7 (4.9)	7 (6.3)
Hispanic	30 (20.8)	27 (24.1)
African	13 (9)	6 (5.4)
South Asian	10 (6.9)	7 (6.3)
European	81 (56.3)	63 (56.3)
Unknown	3 (2.1)	2 (1.8)
Primary cardiac defect, %		
Atrial septal defect (ASD)	33.8	55.7
Ventricular septal defect (VSD)	22.5	17.7
ASD + VSD	13.8	7.6
Atrioventricular canal defect	7.5	6.3
Tetralogy of Fallot	5.6	1.3
Transposition of the great vessels	3.8	3.8
Hypoplastic left heart syndrome	1.3	0
Coarctation of the artery	0.6	0
Other/complex	11.3	7.6

^a^Fisher’s exact test, *p* = 0.009, indicating a higher female-to-male ratio in adult-onset cases compared to pediatric-onset cases

### Rare deleterious variants in known PAH and CHD risk genes

We screened for rare, predicted deleterious variants in 11 known risk genes for PAH and 253 candidate risk genes for CHD (Additional file 1: Table S1). PAH risk gene variants were identified in only 6.4% (16/250) of sporadic PAH-CHD cases and four of 15 familial cases (Additional file 1: Table S2). Of these cases, the majority had pediatric-onset disease (17/144 pediatric vs 3/112 adult, *p* = 0.0085 Fisher’s exact test). Most of the rare deleterious variants were identified in *BMPR2* (*n* = 7, 6 pediatric) and *TBX4* (*n* = 7, all pediatric) with a few variants in *BMPR1A* (n=1), *BMPR1B* (1), *CAV1* (1), *ENG* (1), and *SMAD9* (2). Parental DNA samples were available for a subset of the cases and three *TBX4* variants were confirmed to be de novo: c.C293G:p.P98R, c.537_546del:p.1801 fs*45, and c.669_671del:p.223_224delF. We performed enrichment analysis for the PAH gene set in all PAH-CHD individuals of European ancestry (*n* = 143), using NFE gnomAD WGS subjects (*n* = 7509) as population controls. Similar frequencies of synonymous variants in cases and controls indicated that potential batch effects were minimal between the two independent datasets (Additional file 1: Table S3a). For the known PAH gene set, we observed a 5.7-fold enrichment of rare deleterious (LGD or D-mis) variants in PAH-CHD (*P* = 0.001) (Additional file 1: Table S3b). In contrast, there was no enrichment of rare deleterious variants in CHD risk genes in cases compared to controls (Additional file 1: Table S3b; Additional file 2: Table S4), indicating that overall these variants contribute little to PAH-CHD risk.

### Association analysis identifies transcription factor *SOX17* as a new candidate PAH-CHD risk gene

**Fig. 1:**
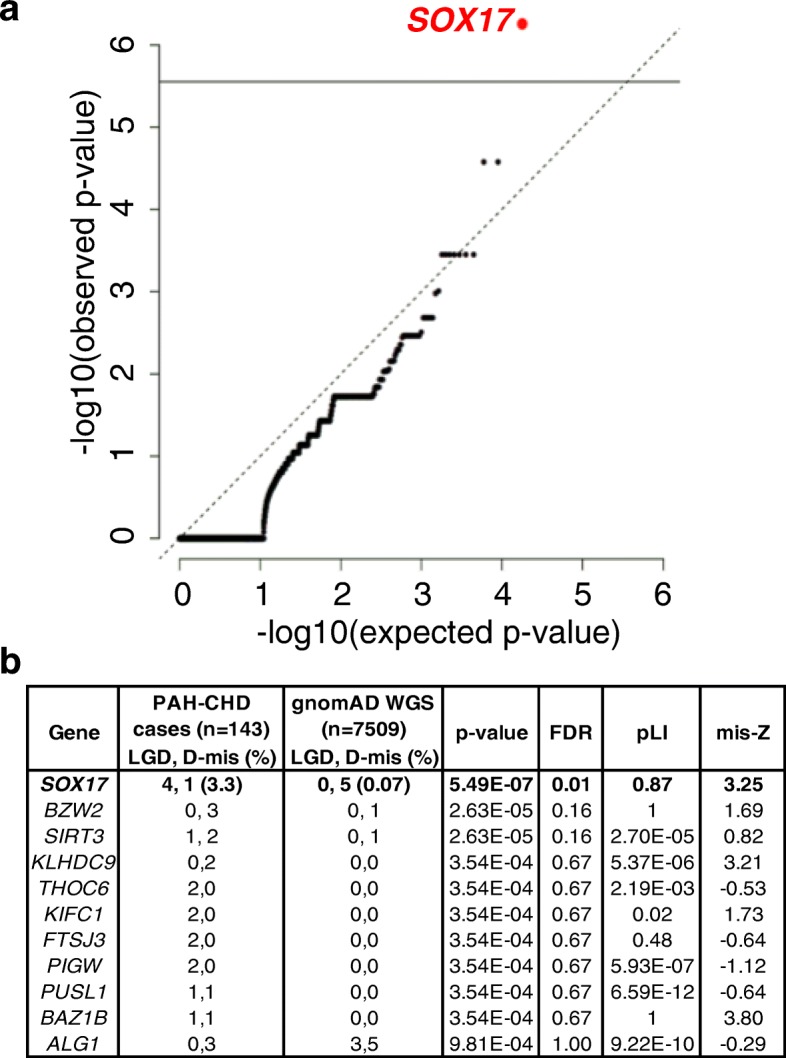
Significant association of *SOX17* with PAH-CHD. **a** Quantile-quantile plot showing results of test of rare variant association in 17,701 genes, using 143 cases of European ancestry and 7509 gnomAD whole genome sequencing subjects of non-Finnish European ancestry. The association of *SOX17* is genome-wide significant following Bonferroni correction for multiple testing. **b** Table of all genes with *p* value < 0.001 in the association tests. False discovery rate (FDR) was estimated using Benjamini-Hochberg procedure. LGD, likely gene-disrupting; D-mis, damaging missense defined as REVEL score > 0.5

To identify novel risk genes for PAH-CHD, we performed an association analysis comparing per-gene rate of rare deleterious variants in European cases and NFE gnomAD WGS controls. We used a binomial test to assess the significance in 17,701 genes and found *SOX17* to be associated with PAH-CHD with genome-wide significance (5/143, 3.3% of cases vs 5/7509, 0.07% of controls; enrichment rate = 52, *p* value = 5.5e−07) (Fig. 1). Analysis of the depth of coverage in the targeted *SOX17* region indicated nearly 100% of gnomAD samples and a slightly lower percentage of PAH-CHD samples attained read depths of at least 10 (Additional file 1: Figure S2), excluding the possibility that the association is driven by coverage difference between cases and population data. No other genes reached the threshold for genome-wide significance. The top associations with a Benjamini-Hochberg FDR < 1.0 are listed in Fig. 1b. Notably, three of these genes (*BZW2*, *FTSJ3*, *BAZ1B*) encode putative SOX17 downstream targets [38] and two have been implicated in CHD (*BAZ1B* [39]) or cardiac defects associated with syndromic intellectual ability (*THOC3* [40]). Similar results were obtained using a smaller cohort of European controls with individual-level data, called and annotated together with the PAH-CHD cases (Additional file 1: Figure S3). Based on the different frequencies between cases and population controls, we estimate that rare deleterious variants in *SOX17* contribute to about 3.2% of European PAH-CHD patients.

**Fig. 2:**
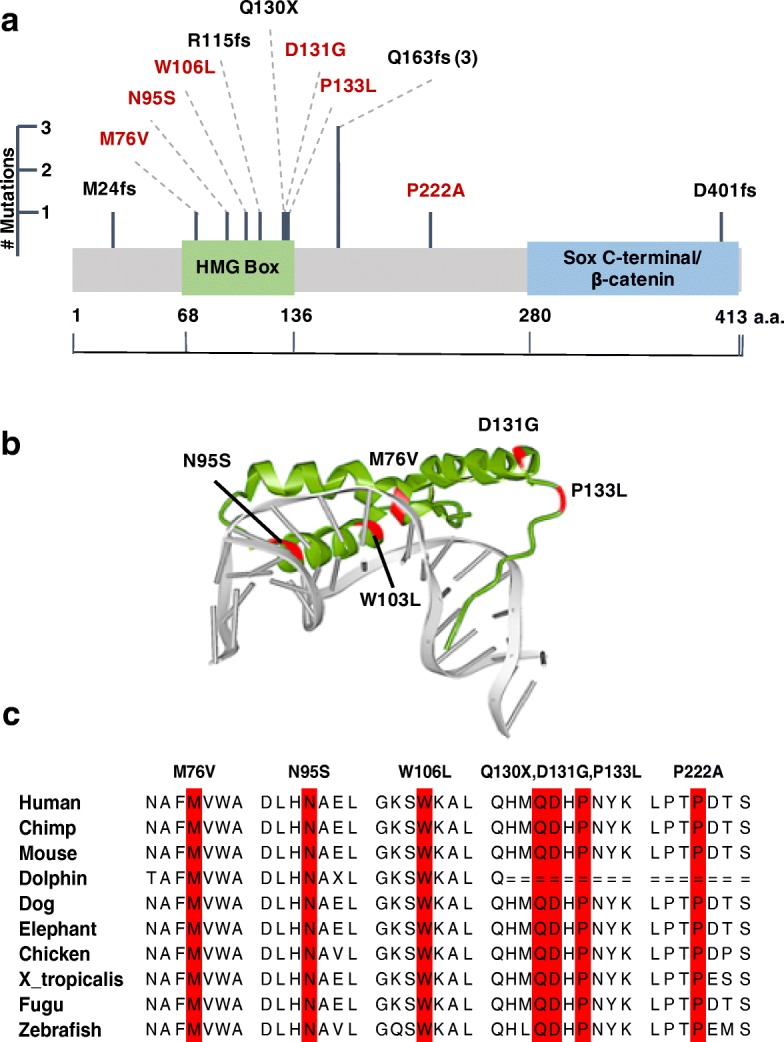
Rare deleterious variants in *SOX17*. **a** Linear schematic of the *SOX17* encoded protein and location of genetic variants identified by WES. LGD variants are in black, D-mis variants in red. **b** Three-dimensional structure of the SOX17 HMG box domain, comprised of three alpha-helices, bound to the minor groove of DNA (Protein Data Bank 3F27). Localization of the five patient D-mis variants (red) indicates that three reside within the DNA binding pocket. **c** Multiple sequence alignment indicating a high degree of sequence conservation across species at the locations of *SOX17* missense variants

We then searched for *SOX17* variants in the non-European cases in the PAH-CHD cohort, and an additional cohort of 413 idiopathic and familial PAH patients without CHD (IPAH/HPAH) [41]. We identified two additional rare LGD and three additional rare D-mis variants in the PAH-CHD cohort, and one additional rare LGD (Table 2) and two rare D-mis variants in the IPAH/HPAH cohort. Variant c.C398T:p.133L, from a European patient, was not included in the initial association analysis due to in silico quality control failure but was later confirmed by Sanger sequencing. Frameshift variant c.489_510del/ p.Q163fs was observed in three unrelated patients of European or Hispanic ancestry. Closer examination of the sequence revealed a 10-bp repeat, once at the start of the deletion and once just downstream (data not shown), suggesting that a replication error may explain the recurrence. Among these three c.489_510del/p.Q163fs mutations, one was a de novo variant and another inherited from an asymptomatic parent (Table 2). Five of the six missense mutations occur within a highly conserved DNA-binding HMG-box domain (Fig. 2a). Three-dimensional modeling indicates that three of these mutations (M76V, N95S, W106L) localize within the DNA binding pocket (Fig. 2b). Comparative sequence analysis shows that all six of the missense variants are in sites highly conserved between species, including vertebrates and invertebrates (Fig. 2c).

**Table 2:** Rare deleterious *SOX17* variants identified in 258 PAH-CHD and 413 IPAH/HPAH samples

Proband ID	Gender	Age at dx (years)	Disease class	Heart defect^a^	Ancestry	*SOX17* exon^b^	Nucleotide change	AA change	Inheritance	Allele frequency (gnomAD)	CADD	REVEL score^c^
JM0016	M	5	PAH-CHD	ASD	European	2	c.C398T	p.P133L	Paternal	–	32.0	0.91
JM0025	M	7 months	PAH-CHD	VSD	European	2	c.489_510del	p.Q163fs	De novo	–	33	N/A
JM1277	F	30	PAH-CHD	ASD	Asian	2	c.1203delC	p.D401fs	Unknown	–	24.1	N/A
JM1417	F	3	PAH-CHD	ASD	European	2	c.489_510del	p.Q163fs	Paternal or de novo	–	33	N/A
JM174	F	14	PAH-CHD	ASD	European	2	c.344delG	p.R115fs	Maternal	–	35	N/A
JM654	M	1	PAH-CHD	PDA	Hispanic	1	c.A284G	p.N95S	Unknown	–	24.7	0.93
JM673	M	34	PAH-CHD	ASD	European	2	c.C388T	p.Q130X	Unknown	–	39.0	N/A
JM887	F	3	PAH-CHD	PDA	European	1	c.A226G	p.M76V	Unknown	–	28.7	0.97
JM951	M	9	PAH-CHD	ASD, VSD, AV canal defect, sinus inversus, mitral cleft	Hispanic	2	c.C664G	p.P222A	Unknown	–	26.1	0.57
SPH1070EW5480	F	38	PAH-CHD	Unknown	Hispanic	2	c.A392G	p.D131G	Unknown	–	22.4	0.89
SPH831KB5173	F	32	IPAH	N/A	European	2	c.G317T	p.W106L	Unknown	–	28.4	0.9
JM1363	F	5	IPAH	N/A	Hispanic	2	c.489_510del	p.Q163fs	Maternal	–	33	N/A
FPPH126-01	M	3	HPAH	N/A	European	1	c.72_76del	p.M24fs	Unknown	–	33	N/A

^a^ASD, atrial septal defect; PDA, patent ductus arteriosus; VSD, ventricular septal defect; AV, atrioventricular

^b^*SOX17* variants identified from transcript NM_022454

^c^Rare, deleterious variants defined as gnomAD AF < 0.01% and REVEL > 0.5

We hypothesized that deleterious variants in *SOX17* confer PAH-CHD risk through dysregulation of SOX17 target genes and some of these genes may contribute to PAH-CHD risk directly, independent of *SOX17*. Therefore, we tested for enrichment of rare variants in 1947 putative *SOX17* target genes identified by genome-wide ChIP-X experiments [38] in European cases compared to NFE gnomAD WGS subjects. We observed a moderate but significant enrichment of rare missense variants (enrichment rate = 1.16, *p* value = 3.4e−4) (Additional file 1: Table S5). Since there are 618 rare missense variants in these genes in 143 cases, even a moderate enrichment suggests a large number of rare variants in *SOX17*-regulated genes may contribute to PAH-CHD risk. Using publicly available gene expression data for developing heart [17] and adult pulmonary artery endothelial cells (ENCODE RNA-seq data, ENCBS024RNA), we found that the majority of the SOX17 target genes with rare deleterious variants are expressed in one or both of these tissue/cell types, with 28% (42/149) having top quartile expression in *both* tissue/cell types (Additional file 1: Table S6 and Fig. S4a). We assessed the statistical significance of this expression pattern by building a background distribution with randomly selected sets of 149 genes that carry at least one rare LGD or D-mis variant in cases and counted the number of genes with top quartile ranked expression in both tissues. Based on 100,000 simulations, the number of observed genes in the top quartile of developing heart and PAEC expression in the SOX17 targets (42 out of 149) is significantly larger than expectation by chance (*p* ≤ 10–5) (Additional file 1: Figure S4b), supporting functional relevance of these SOX17 target genes. Pathway enrichment analysis using Reactome 2016 [42, 43] through Enrichr (amp.pharm.mssm.edu/Enrichr/enrich) showed that the SOX17 target genes with deleterious variants are over-represented (FDR-adjusted *p* value < 0.05) in (1) developmental processes, (2) transmembrane transport of small molecules and ion homeostasis, and (3) extracellular matrix interactions (Additional file 1: Table S7).

### Contribution of de novo mutations to PAH-CHD

We have previously reported an enrichment of de novo predicted deleterious variants in a CHD cohort ascertained without considering PAH [17, 18]. We tested for a role of de novo mutations in PAH-CHD in 60 cases with WES data of biological parents (“trios”). The complete list of 60 rare de novo variants is provided in Additional file 1: Table S8. As mentioned previously, three de novo variants were identified in PAH risk gene *TBX4* and one variant each in CHD risk genes *NOTCH1* and *PTPN11*. However, testing for enrichment of all rare de novo variants in PAH-CHD trio probands compared to an estimated background mutation rate indicated no overall enrichment, likely due to the small sample size.

## Discussion

Exome sequencing in our cohort of 256 PAH-CHD patients indicated that the genetic contribution of known/candidate risk genes for PAH or CHD alone is minimal. An unbiased, gene-based association analysis of rare deleterious variants identified *SOX17* as a novel PAH-CHD candidate risk gene, explaining up to 3.2% of cases. A recent study of 1038 PAH cases (not including PAH-CHD) also found an association of *SOX17* with IPAH but with a smaller effect size (relative risk ~ 2.9) [44]. The observed frequency of rare variants was ~ 0.9% of PAH cases [44], similar to our observation of *SOX17* variants in ~ 0.7% of IPAH/HPAH patients without CHD. Of note, no rare deleterious *SOX17* variants were identified in a recently published cohort of 1200 patients with CHD [18]. Additionally, we observed an enrichment of rare variants in putative target genes of SOX17. There was no enrichment of de novo mutations in this cohort, possibly due to the relatively small number of available trios.

*SOX17* is a member of the conserved *SOX* family of transcription factors widely expressed in development, and the subgroup of *SOXF* genes (including *SOX7*, *SOX17*, and *SOX18*) participate in vasculogenesis and remodeling [45]. In the embryonic vasculature, *SOX17* is selectively expressed in arterial endothelial cells [46–48]. Early studies of *Sox17* knock-out mice did not find obvious abnormalities in embryonic vasculature [49, 50], at least partially explained by functional redundancy and compensatory roles of *Sox17* and *Sox18* [50, 51]. Subsequent genetic studies revealed that gene compensation and phenotypic effects were dependent on strain background [52]. Recent endothelial-specific inactivation of *Sox17* in murine embryo or postnatal retina led to impaired arterial specification and embryonic death or arterial-venous malformations, respectively [46]. *SOX17* has also been associated with intracranial aneurysms in genome-wide association studies [53–55], and endothelial-specific *Sox17* deficiency was subsequently shown to induce intracranial aneurysm pathology in an angiotensin II infusion mouse model [56]. Finally, conditional deletion of *Sox17* in mesenchymal progenitor cells demonstrated that SOX17 is required for normal pulmonary vasculature morphogenesis in utero and deficiency results in postnatal cardiac defects [57].

Cardiogenesis occurs in a highly conserved and regulated manner in the developing embryo [58]. Precise temporal and spatial control of gene expression is controlled by master transcription factors such as GATA4, MEF2C, TBX5, and NKX2–5 [59], In addition, signaling pathways, including canonical and non-canonical WNT/β-catenin [60, 61] and NOTCH [62] signaling cascades, drive cardiac morphogenesis and differentiation. *SOX17* is a direct transcriptional target of GATA4, giving rise to SOX17-positive endoderm from embryonic stem cells [63] and the two proteins co-localize in the primitive endoderm [64, 65]. *SOX17* induction inhibits WNT/β-catenin signaling by direct protein interaction with β-catenin through a carboxyl terminal domain of SOX17 required for transactivation of target genes [66, 67]. *NOTCH1* has recently been shown to be a direct transcriptional target of SOX17 in early arterial development [68]. Thus, it is possible that impaired functional interactions between these molecules during embryogenesis could provide an underlying mechanism for the development of CHD in some PAH-CHD patients.

*SOX17* is a highly constrained gene depleted of LGD and missense variants in a large population data set (ExAC pLI = 0.87, missense Z-score = 3.25) [26]. About half of the observed rare, deleterious variants in cases are LGD variants, and most of the missense variants are located in a conserved HMG box domain. The HMG box is a 79-amino acid domain that binds in a sequence-specific manner within the minor groove of DNA causing bending and facilitating assembly of nucleoprotein complexes [45]. Localization of the five HMG box missense variants within a three-dimensional model of the protein domain interacting with DNA indicated that three of the patient missense mutations (M76V, N95S, W106L) localize to the DNA binding pocket (Fig. 2b). Previously reported site-directed mutagenesis studies indicate that similar point mutations within this region (M76A, G103R) can impair both direct DNA binding [69] and complex nucleoprotein interactions, including SOX17/β-catenin protein complexes, at target gene promoters [70, 71]. This suggests that haploinsufficiency with loss of function alleles is the likely mechanism of *SOX17* risk in PAH-CHD.

Some variants in SOX17 downstream target genes may be predicted to mimic some of the consequences of *SOX17* loss of function mutations or haploinsufficiency. We identified 163 rare deleterious variants (131 D-mis and 32 LGD) in 149 putative target genes. Using published gene expression data, we found that most of these genes are expressed in developing heart and/or pulmonary artery endothelial cells, with significant enrichment of top quartile expression in both tissue/cell types compared to randomly selected sets of genes carrying deleterious variants in European PAH-CHD cases. Additionally, we showed that these target genes are overrepresented in pathways related to developmental biology, ion transport/homeostasis, and extracellular matrix interactions. A wide range of transmembrane small molecule transporters/channels/pumps are expressed in developing heart and pulmonary vasculature, and some have been shown to be differentially expressed in lung tissue from PAH patients compared to non-disease controls or PH with interstitial fibrosis [72]. As key regulators of vascular tone, some of these molecules function as targets of vasodilatory pharmacotherapy [73]. We recently identified the potassium channel gene, *KCNK3*, as a risk gene for PAH using exome sequencing [74]. Extracellular matrix proteins, including laminins, play key roles in embryonic development of both pulmonary vasculature and heart [75]. Thus, it is likely that mutations in *SOX17*, and possibly downstream target genes, may increase risk for PAH-CHD via multiple pathways.

The striking clinical finding was that nine out of 13 patients had pediatric-onset disease. The mean age of PAH onset for all patients with rare *SOX17* variants was 14.2 years. Most of the congenital heart defects were simple (i.e., atrial septal defect, ventricular septal defect, or patent ductus arteriosus). However, most of the patients had severe PAH with systemic or supersystemic resting pulmonary arterial pressures, right ventricular hypertrophy with diminished right ventricular function, and requiring chronic intravenous vasodilator treatment. Severe PAH was observed in all patients carrying variants in the HMG-box domain or the recurrent c.489_510del/ p.Q163fs variant.

## Conclusions

Together, these data strongly implicate *SOX17* as a new risk gene contributing to ~ 3% of PAH-CHD cases and suggest that rare variants in genes regulated by SOX17 also contribute to PAH-CHD. Expansion of the number of PAH-CHD patients assessed and characterization of the clinical phenotypes will be important to confirm the role of *SOX17* in PAH-CHD and IPAH, and more precisely estimate the contribution of genes regulated by SOX17 and de novo mutations.

## Additional files

Additional file 1: **Figure S1.** Study overview. **Figure S2.** Depth of sequencing coverage for SOX17. **Figure S3.** Gene-based association analysis using in-house controls. **Figure S4.** SOX17 target gene expression in murine E14.5 developing heart and human adult pulmonary aortic endothelial cells. **Figure S5.** Gene ontology analysis of SOX17 target genes harboring PAH-CHD patient-derived rare deleterious variants. **Table S1.** List of known PAH and CHD candidate risk genes. **Table S2.** Variants in known PAH risk genes. **Table S3.** Enrichment analyses in European cases and controls. **Table S5.** Enrichment analysis for SOX17 target genes. **Table S6.** SOX17 target gene variants and gene expression rank. **Table S7.** De novo variants. **Table S8.** List of all rare de novo variants in pediatric-onset PAH-CHD trios (*n*=60). (DOCX 2.05 mb)

Additional file 2: **Table S4.** Variants in known CHD risk genes. (XLSX 46 kb)

## Abbreviations

*ACVRL1*: Activin A receptor-like 1
AF: Allele frequency
*BMPR1A*: Bone morphogenetic protein receptor type 1A
*BMPR1B*: Bone morphogenetic protein receptor type 1B
*BMPR2*: Bone morphogenetic protein receptor type 2
bp: Base pair
BWA-MEM: Burrows-Wheeler Aligner
CADD: Combined Annotation Dependent Depletion
*CAV1*: Caveolin-1
CHD: Congenital heart disease
D-mis: Damaging missense variants
*EIF2AK4*: Eukaryotic initiation translation factor 2 alpha kinase 4
*ENG*: Endoglin
ExAC: Exome Aggregation Consortium
FDR: False discovery rate
gnomAD: Genome Aggregation Database
IGV: Integrative Genomics Viewer
IPAH: Idiopathic pulmonary arterial hypertension
*KCNK3*: Potassium two-pore-domain channel subfamily K member 3
LGD: Likely gene-disrupting
MLPA: Multiplex ligation-dependent probe amplification
NFE: Non-Finnish Europeans
*NOTCH1*: Notch (Drosophila) homolog 1
PAH: Pulmonary arterial hypertension
PAH-CHD: Pulmonary arterial hypertension associated with congenital heart disease
PCA: Principal components analysis
*PTPN11*: Protein tyrosine phosphatase non-receptor type 11
REVEL: Rare exome variant ensemble learner
RGC: Regeneron Genetics Center
SMAD4: SMAD family member 4
SMAD9: SMAD family member 9
*SOX17*: SRY-related HMG-box family member 17
*TBX4*: T-box 4
TGF-β/BMP: Transforming growth factor beta/bone morphogenetic protein
VQSR: Variant quality score recalibration
WES: Whole exome sequencing
WGS: Whole genome sequencing
WHO: World Health Organization
