# Supplementary material for: Rare variants in SOX17 are associated with pulmonary arterial hypertension with congenital heart disease
Source: Genome Med. 2018 Jul 20;10:56. doi: 10.1186/s13073-018-0566-x (PMC6054746; doi:10.1186/s13073-018-0566-x)
Supplement: Supplementary file 1 — Figure S1. Study overview. Figure S2. Depth of sequencing coverage for SOX17. Figure S3. Gene-based association analysis using in-house controls. Figure S4. SOX17 target gene expression in murine E14.5 developing heart and human adult pulmonary aortic endothelial cells. Figure S5. Gene ontology analysis of SOX17 target genes harboring PAH-CHD patient-derived rare deleterious variants. Table S1. List of known PAH and CHD candidate risk genes. Table S2. Variants in known PAH risk genes. Table S3. Enrichment analyses in European cases and controls. Table S5. Enrichment analysis for SOX17 target genes. Table S6. SOX17 target gene variants and gene expression rank. Table S7. De novo variants. Table S8. List of all rare de novo variants in pediatric-onset PAH-CHD trios (n=60). (DOCX 2.05 mb) [file 13073_2018_566_MOESM1_ESM.docx]

**Figure S1. Study overview.** **a)** Work-flow chart. **b)** Comparison of multiple deleterious prediction tools indicating that REVEL is a better predictor of likely deleterious missense variants. **c)** Principle components analysis (PCA) of ethnicity using *Peddy* (<https://github.com/brentp/peddy>). Europeans localized to a discrete cluster for both cases (purple squares) and 1000 genomes controls (purple dots).

**Figure S2. Depth of sequencing coverage for *SOX17*.** Comparison of PAH-CHD samples and gnomAD WGS controls at read depth (DP) ≥10 or ≥15.

**Figure S3. Gene-based association analysis using 143 European PAH-CHD cases and 1319 European PCGC controls. a)** Principle components analysis of ethnicity using *Peddy*. Europeans localized to a discrete cluster (purple) for both PCGC controls and PAH-CHD cases. **b)** Results of a binomial test confined to rare LGD and D-mis (REVEL >0.5) variants. The top association signal was for *SOX17* (p=9.15e-05). **c**. Complete list of top association genes (p<=0.001); genes in bold-face were also among the top associations using gnomAD controls.

**c.**

| Gene | #Variants, cases  (LGD+D-mis) | #Variants, control  (LGD+D-mis) | P-value | OR |
| --- | --- | --- | --- | --- |
| ***SOX17*** | **4** | **0** | **9.15E-05** | **Inf** |
| *DMBX1* | 3 | 0 | 9.36E-04 | Inf |
| ***VANGL1*** | **3** | **0** | **9.36E-04** | **Inf** |
| ***SIRT3*** | **3** | **1** | **3.47E-03** | **27.67** |
| ***ALG1*** | **3** | **1** | **3.47E-03** | **27.67** |
| ***ABCA4*** | **5** | **7** | **3.47E-03** | **6.59** |
| *MMEL1* | 3 | 2 | 3.47E-03 | 13.84 |
| *LOXHD1* | 3 | 2 | 3.47E-03 | 13.84 |
| *KLHDC9* | 2 | 0 | 3.47E-03 | Inf |
| *TCP11L1* | 2 | 0 | 3.47E-03 | Inf |
| ***THOC6*** | **2** | **0** | **3.47E-03** | **Inf** |
| *STAP1* | 2 | 0 | 3.47E-03 | Inf |
| ***BAZ1B*** | **2** | **0** | **3.47E-03** | **Inf** |
| *ATL3* | 2 | 0 | 3.47E-03 | Inf |
| *RARG* | 2 | 0 | 3.47E-03 | Inf |
| *PWP2* | 2 | 0 | 3.47E-03 | Inf |
| *PFKFB2* | 2 | 0 | 3.47E-03 | Inf |
| *TPO* | 2 | 0 | 3.47E-03 | Inf |
| *KIT* | 2 | 0 | 3.47E-03 | Inf |
| *PALLD* | 2 | 0 | 3.47E-03 | Inf |
| *RICTOR* | 2 | 0 | 3.47E-03 | Inf |
| ***PELO*** | **2** | **0** | **3.47E-03** | **Inf** |
| ***BZW2*** | **2** | **0** | **3.47E-03** | **Inf** |
| *MUSK* | 2 | 0 | 3.47E-03 | Inf |
| *RDX* | 2 | 0 | 3.47E-03 | Inf |
| *PTGR2* | 2 | 0 | 3.47E-03 | Inf |
| *COG7* | 2 | 0 | 3.47E-03 | Inf |
| *SBNO2* | 2 | 0 | 3.47E-03 | Inf |
| *TJP3* | 2 | 0 | 3.47E-03 | Inf |
| *ADA* | 2 | 0 | 3.47E-03 | Inf |
| *MOB3A* | 2 | 0 | 3.47E-03 | Inf |

**Figure S4. SOX17 target gene expression in murine E14.5 developing heart and human adult pulmonary artery endothelial cells (PAECs). a)** Rank expression of 149 SOX17 target genes with PAH-CHD patient-derived rare deleterious variants. Genes with LGD variants are represented by blue dots; genes with D-mis variants by red dots. b) Null distribution of the number of genes with top quartile expression, in both developing heart and PAECs, in randomly selected sets of 149 genes with at least one rare LGD or D-mis variant in European PAH-CHD cases. 100,000 simulations were performed. The red line shows that 42 of the 149 SOX17 target genes have top quartile expression in both developing heart and PAECs, indicating significant enrichment (p<1.0E-05).

# genes with top quartile expression in developing heart and PAECs

Density

Expression in developing heart

Expression in PAECs

b)

a)


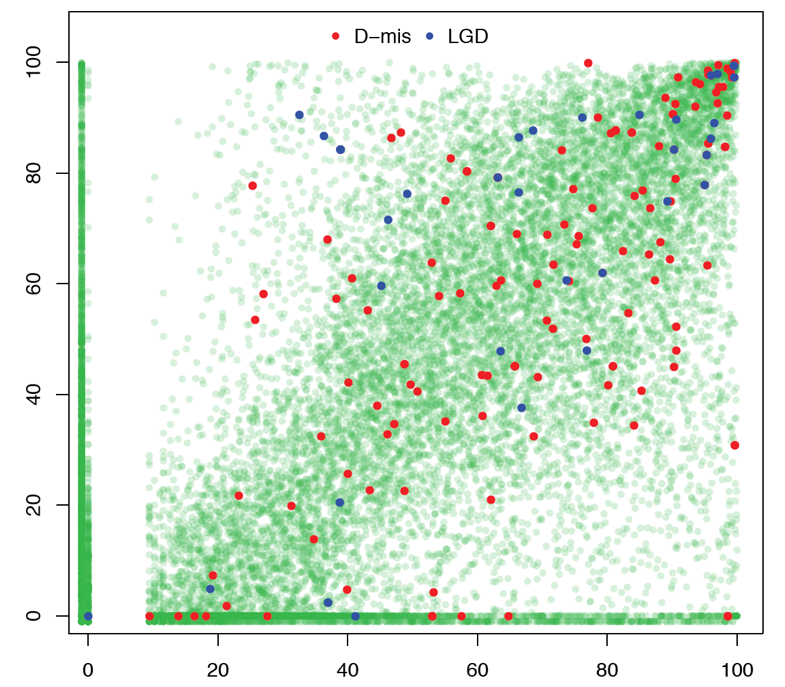


| **Table S1. List of known PAH and CHD risk genes** |  |
| --- | --- |
| **PAH Risk Genes** | **OMIM** |
| *ACVRL1*, activin A, receptor type II- like 1 | 601284 |
| *BMPR1A*, bone morphogenic protein receptor type IA | 601299 |
| *BMPR1B*, bone morphogenic protein receptor type IB | 603248 |
| *BMPR2*, bone morphogenic protein receptor type II | 600799 |
| *CAV1*, caveolin-1 | 601047 |
| *EIF2AK4*, eukaryotic initiation translation factor 2 alpha kinase 4 | 609280 |
| *ENG*, endoglin | 131195 |
| *KCNK3*, potassium two-pore-domain channel subfamily K member 3 | 603220 |
| *SMAD4*, SMAD family member 4 | 600993 |
| *SMAD9*, SMAD family member 9 | 603295 |
| *TBX4*, T-box 4 | 601719 |
| **CHD Risk Genes - Human** | **OMIM** |
| *ABCC9*, ATP Binding Cassette Subfamily C Member 9 | 601439 |
| *ABCD3,* ATP Binding Cassette Subfamily D Member 3 | 170995 |
| *ACTB*, Actin, Beta | 102630 |
| *ACVR2B*, Activin A Receptor, Type IIB | 602730 |
| *ADAMTS10*, ADAM Metallopeptidase With Thrombospondin Type 1 Motif 10 | 608990 |
| *ADNP*, Activity Dependent Neuroprotector Homeobox | 611386 |
| *ANKRD11.* Ankyrin Repeat Domain 11 | 611192 |
| *ARHGAP31*, Rho GTPase Activating Protein 31 | 610911 |
| *ARID1A*, AT-Rich Interaction Domain 1A | 603024 |
| *ARL13B*, ADP Ribosylation Factor Like GTPase 13B | 608922 |
| *ARMC4*, Armadillo Repeat Containing 4 | 615408 |
| *ASXL1*, Additional Sex Combs Like 1, Transcriptional Regulator | 612990 |
| *ATIC,* 5-Aminoimidazole-4-Carboxamide Ribonucleotide Formyltransferase/IMP Cyclohydrolase | 601731 |
| *B3GALT6*, Beta-1,3-Galactosyltransferase 6 | 615291 |
| *BBS1,* Bardet-Biedl Syndrome 1 | 209901 |
| *BBS10*, Bardet-Biedl Syndrome 10 | 610148 |
| *TRIM32*, Tripartite Motif Containing 32 | 602290 |
| *BBS12*, Bardet-Biedl Syndrome 12 | 610683 |
| *BBS2*, Bardet-Biedl Syndrome 2 | 606151 |
| *ARL6*, ADP Ribosylation Factor Like GTPase 6 | 608845 |
| *BBS4,* Bardet-Biedl Syndrome 4 | 615982 |
| *BBS5*, Bardet-Biedl Syndrome 5 | 615983 |
| *MKKS*, McKusick-Kaufman Syndrome | 604896 |
| *BBS7*, Bardet-Biedl Syndrome 5 | 603650 |
| *TTC8*, Tetratricopeptide Repeat Domain 8 | 608132 |
| *BBS9*, Bardet-Biedl Syndrome 5 | 603650 |
| *BCOR,* BCL6 Corepressor | 300485 |
| *BRAF*, B-Raf Proto-Oncogene, Serine/Threonine Kinase | 164757 |
| *CACNA1C*, Calcium Voltage-Gated Channel Subunit Alpha1 C | 114205 |
| *CBL*, Cbl Proto-Oncogene | 165360 |
| *CCDC103,* Coiled-Coil Domain Containing 103 | 614677 |
| *CCDC114*, Coiled-Coil Domain Containing 114 | 615038 |
| *CCDC151*, Coiled-Coil Domain Containing 151 | 615956 |
| *CCDC39*, Coiled-Coil Domain Containing 39 | 613798 |
| *CCDC40*, Coiled-Coil Domain Containing 40 | 613799 |
| *CD96*, CD96 Molecule | 606037 |
| *CDKN1C*, Cyclin Dependent Kinase Inhibitor 1C | 600856 |
| *CEP290,* Centrosomal Protein 290-KD | 610142 |
| *CEP41*, Centrosomal Protein 41-KD | 610523 |
| *CEP57,* Centrosomal Protein 57-KD | 607951 |
| *CFC1*, Cripto, FRL-1, Cryptic Family 1 | 605194 |
| *CHD4*, Chromodomain Helicase DNA Binding Protein 4 | 603277 |
| *CHD7*, Chromodomain Helicase DNA Binding Protein 7 | 608892 |
| *CHST3*, Carbohydrate Sulfotransferase 3 | 603799 |
| *CITED2*, Cbp/P300 Interacting Transactivator With Glu/Asp Rich Carboxy-Terminal Domain 2 | 602937 |
| *COL1A1*, Collagen Type I Alpha 1 Chain | 120150 |
| *COL1A2*, Collagen Type I Alpha 2 Chain | 120160 |
| *COL2A1*, Collagen Type 2 Alpha 1 Chain | 120140 |
| *COL3A1*, Collagen Type 3 Alpha 1 Chain | 120180 |
| *COL5A1*, Collagen Type 5 Alpha 1 Chain | 120215 |
| *COL5A2*, Collagen Type 5 Alpha 2 Chain | 120190 |
| *COX7B*, Cytochrome C Oxidase Subunit 7B | 300885 |
| *CREBBP*, CREB Binding Protein | 600140 |
| *CRELD1*, Cysteine Rich With EGF Like Domains 1 | 607170 |
| *DDX11*, DEAD/H-Box Helicase 11 | 601150 |
| *DGCR2*, DiGeorge Syndrome Critical Region Gene 2 | 600594 |
| *DHCR7*, 7-Dehydrocholesterol Reductase | 602858 |
| *DNAAF1*, Dynein Axonemal Assembly Factor 1 | 613190 |
| *DNAAF2*, Dynein Axonemal Assembly Factor 2 | 612517 |
| *DNAAF3*, Dynein Axonemal Assembly Factor 3 | 614566 |
| *DNAAF4/DYX1C1*, Dynein Axonemal Assembly Factor 4 | 608706 |
| *DNAH11*, Dynein Axonemal Heavy Chain 11 | 603339 |
| *DNAH5*, Dynein Axonemal Heavy Chain 5 | 603335 |
| *DNAI1*, Dynein Axonemal Intermediate Chain 1 | 604366 |
| *DNAI2*, Dynein Axonemal Intermediate Chain 2 | 605483 |
| *DNAL1*, Dynein Axonemal Light Chain 1 | 610062 |
| *DOCK6*, Dedicator Of Cytokinesis 6 | 614194 |
| *DYNC2H1*, Dynein Cytoplasmic 2 Heavy Chain 1 | 603297 |
| *ECE1*, Endothelin Converting Enzyme 1 | 600423 |
| *EFTUD2*, Elongation Factor Tu GTP Binding Domain Containing 2 | 603892 |
| *EHMT1*, Euchromatic Histone Lysine Methyltransferase 1 | 607001 |
| *ELN*, Elastin | 130160 |
| *EOGT*, EGF Domain Specific O-Linked N-Acetylglucosamine Transferase | 614789 |
| *ESCO2*, Establishment Of Sister Chromatid Cohesion N-Acetyltransferase 2 | 609674 |
| *EVC*, EvC Ciliary Complex Subunit 1 | 604831 |
| *EVC2*, EvC Ciliary Complex Subunit 2 | 607261 |
| *FBN1*, Fibrillin 1 | 134797 |
| *FBN2*, Fibrillin 2 | 612570 |
| *FGF8*, Fibroblast Growth Factor 8 | 600483 |
| *FGFR1*, Fibroblast Growth Factor Receptor 1 | 136350 |
| *FIG4*, FIG4 Phosphoinositide 5-Phosphatase | 609390 |
| *FKTN*, Fukutin | 607440 |
| *FLNA*, Filamin A | 300017 |
| *FOXC1*, Forkhead Box C1 | 601090 |
| *FOXC2*, Forkhead Box C2 | 602402 |
| *FTO*, Alpha-Ketoglutarate Dependent Dioxygenase | 610966 |
| *GATA4*, GATA Binding Protein 4 | 600576 |
| *GATA6*, GATA Binding Protein 6 | 601656 |
| *GDF1*, Growth Differentiation Factor 1 | 602880 |
| *GJA1*, Gap Junction Protein Alpha 1 | 121014 |
| *GLI3*, GLI Family Zinc Finger 3 | 165240 |
| *GPC3*, Glypican 3 | 300037 |
| *GPC6*, Glypican 6 | 604404 |
| *HCCS*, Holocytochrome C Synthase | 300056 |
| *HOXA1*, Homeobox A1 | 142955 |
| *HRAS*, HRas Proto-Oncogene, GTPase | 190020 |
| *IFT122*, Intraflagellar Transport 122 | 606045 |
| *IFT140*, Intraflagellar Transport 140 | 614620 |
| *IFT80*, Intraflagellar Transport 80 | 611177 |
| *INVS*, Inversin | 243305 |
| *IRX5*, Iroquois Homeobox 5 | 606195 |
| *JAG1*, Jagged 1 | 601920 |
| *C5ORF42*, Chromosome 5 Open Reading Frame 42 | 614571 |
| *KANSL*1, KAT8 Regulatory NSL Complex Subunit 1 | 612542 |
| *KAT6B*, Lysine Acetyltransferase 6B | 605880 |
| *KIF7*, Kinesin Family Member 7 | 611254 |
| *KMT2A*, Lysine Methyltransferase 2A | 159555 |
| *KMT2D*, Lysine Methyltransferase 2D | 602113 |
| *KRAS*, KRAS Proto-Oncogene, GTPase | 190070 |
| *LBR*, Lamin B Receptor | 600024 |
| *LEFTY2*, Left-Right Determination Factor 2 | 601877 |
| *LRP2*, LDL Receptor Related Protein 2 | 600073 |
| *LTBP4*, Latent Transforming Growth Factor Beta Binding Protein 4 | 604710 |
| *MAP2K1*, Mitogen-Activated Protein Kinase Kinase 1 | 176872 |
| *MAP2K2*, Mitogen-Activated Protein Kinase Kinase 2 | 601263 |
| *MED12*, Mediator Complex Subunit 12 | 300188 |
| *MED13L, Mediator Complex Subunit 13-like* | 608771 |
| *MEGF8, Multiple EGF Like Domains 8* | 604267 |
| *MGP*, Matrix Gla Protein | 154870 |
| *MID1*, Midline 1 | 300552 |
| *MKS1*, Meckel Syndrome, Type 1 | 249000 |
| *MYH6*, Myosin Heavy Chain 6 | 160710 |
| *NEK1*, NIMA Related Kinase 1 | 604588 |
| *NF1*, Neurofibromatosis Type 1 | 162200 |
| *NIPBL*, NIPBL, Cohesin Loading Factor | 608667 |
| *NKX2-5*, NK2 Homeobox 5 | 600584 |
| *NKX2-6*, NK2 Homeobox 6 | 603245 |
| *NME8,* NME/NM23 Family Member 8 | 607421 |
| *NODAL*, Nodal Growth Differentiation Factor | 601265 |
| *NOTCH1*, Notch1 | 190198 |
| *NOTCH2*, Notch2 | 600275 |
| *NPHP3*, Nephrocystin 3 | 608002 |
| *NPHP4*, Nephrocystin 4 | 607215 |
| *NEK8*, NIMA Related Kinase 8 | 609799 |
| *NR2F2*, Nuclear Receptor Subfamily 2 Group F Member 2 | 107773 |
| *NRAS*, NRAS Proto-Oncogene, GTPase | 164790 |
| *NSD1*, Nuclear Receptor Binding SET Domain Protein 1 | 606681 |
| *NSDHL*, NAD(P) Dependent Steroid Dehydrogenase-Like | 300275 |
| *OFD1*, OFD1, Centriole And Centriolar Satellite Protein | 300170 |
| *PEX1*, Peroxisomal Biogenesis Factor 1 | 602136 |
| *PEX13*, Peroxisomal Biogenesis Factor 13 | 601789 |
| *PHGDH*, Phosphoglycerate Dehydrogenase | 606879 |
| *PITX2*, Paired Like Homeodomain 2 | 601542 |
| *PKD1*, Polycystin 1 | 601313 |
| *PKD2*, Polycystin 2 | 173910 |
| *PLOD1*, Procollagen-Lysine,2-Oxoglutarate 5-Dioxygenase 1 | 153454 |
| *PKD1L1*, Polycystin 1 Like 1, Transient Receptor Potential Channel Interacting | 609721 |
| *PQBP1*, Polyglutamine Binding Protein 1 | 300463 |
| *PTEN*, Phosphatase And Tensin Homolog | 601728 |
| *PTPN11*, Protein Tyrosine Phosphatase, Non-Receptor Type 11 | 176876 |
| *RAB23*, RAB23, Member RAS Oncogene Family | 606144 |
| *RAD21*, RAD21 Cohesin Complex Component | 606462 |
| *RAF1*, Raf-1 Proto-Oncogene, Serine/Threonine Kinase | 164760 |
| *RAI1*, Retinoic Acid Induced 1 | 607642 |
| *RBM10*, RNA Binding Motif Protein 10 | 300080 |
| *RBM8A*, RNA Binding Motif Protein 8A | 605313 |
| *RIT1*, Ras Like Without CAAX 1 | 609591 |
| *RNU4ATAC*, RNA, U4atac Small Nuclear (U12-Dependent Splicing) | 601428 |
| *ROR2*, Receptor Tyrosine Kinase Like Orphan Receptor 2 | 602337 |
| *RPGRIP1L,* RPGRIP1-like | 610937 |
| *RPL11*, Ribosomal Protein L11 | 604175 |
| *RPL35A*, Ribosomal Protein L35A | 180468 |
| *RPL5*, Ribosomal Protein L5 | 603634 |
| *RPS10*, Ribosomal Protein S10 | 603632 |
| *RPS17*, Ribosomal Protein S17 | 180472 |
| *RPS19*, Ribosomal Protein S19 | 603474 |
| *RPS24*, Ribosomal Protein S24 | 602412 |
| *RPS26*, Ribosomal Protein S26 | 603701 |
| *RPS7*, Ribosomal Protein S7 | 603658 |
| *RSPH4A*, Radial Spoke Head 4 Homolog A | 612647 |
| *RSPH9*, Radial Spoke Head 4 Homolog H9 | 612648 |
| *SALL1*, Spalt Like Transcription Factor 1 | 602218 |
| *SEMA3E*, Semaphorin 3E | 608166 |
| *SETBP1*, SET Binding Protein 1 | 611060 |
| *SF3B4*, Splicing Factor 3b Subunit 4 | 605593 |
| *SH3PXD2B*, SH3 And PX Domains 2B | 613293 |
| *SHH*, Sonic Hedgehog | 600725 |
| *SHOC2*, SHOC2, Leucine Rich Repeat Scaffold Protein | 602775 |
| *SKI*, SKI Proto-Oncogene | 164780 |
| *SMAD3*, SMAD Family Member 3 | 603109 |
| *SMAD4*, SMAD Family Member 4 | 600993 |
| *SMARCA4*, SWI/SNF Related, Matrix Associated, Actin Dependent Regulator Of Chromatin, Subfamily A, Member 4 | 603254 |
| *SMARCB1,* SWI/SNF Related, Matrix Associated, Actin Dependent Regulator Of Chromatin, Subfamily B, Member 1 | 601607 |
| *SMARCE1*, SWI/SNF Related, Matrix Associated, Actin Dependent Regulator Of Chromatin, Subfamily E, Member 1 | 603111 |
| *SMC3*, Structural Maintenance Of Chromosomes 3 | 606062 |
| *SMS*, Spermine Synthase | 182290 |
| *SOS1*, SOS Ras/Rac Guanine Nucleotide Exchange Factor 1 | 182530 |
| *SOX2*, SRY-Box 2 | 184429 |
| *SOX9*, SRY-Box 9 | 608160 |
| *STAMBP*, STAM Binding Protein | 606247 |
| *STRA6*, Stimulated by Retinoic Acid 6 | 610745 |
| *TAB2*, TAK1 Binding Protein 2 | 605101 |
| *TBX1*, T-Box 1 | 602054 |
| *TBX20*, T-Box 20 | 606061 |
| *TBX3*, T-Box 3 | 601621 |
| *TBX5*, T-Box 5 | 601620 |
| *TCOF1*, Treacle Ribosome Biogenesis Factor 1 | 606847 |
| *TFAP2B*, Transcription Factor AP-2 Beta | 601601 |
| *TGFBR1*, Transforming Growth Factor Beta Receptor 1 | 190181 |
| *TGFBR2*, Transforming Growth Factor Beta Receptor 2 | 190182 |
| *TLL1*, Tolloid Like 1 | 606742 |
| *TSC1*, Tuberous Sclerosis 1 | 605284 |
| *TSC2*, Tuberous Sclerosis 2 | 191092 |
| *TTC21B*, Tetratricopeptide Repeat Domain 21B | 612014 |
| *TWIST1*, Twist Family BHLH Transcription Factor 1 | 601622 |
| *UBR1*, Ubiquitin Protein Ligase E3 Component N-Recognin 1 | 605981 |
| *WDR19*, WD Repeat Domain 19 | 608151 |
| *WDR35*, WD Repeat Domain 35 | 613602 |
| *WDR60*, WD Repeat Domain 60 | 615462 |
| *ZEB2*, Zinc Finger E-Box Binding Homeobox 2 | 605802 |
| *ZFPM2*, Zinc Finger Protein, FOG Family Member 2 | 603693 |
| *ZIC3*, Zic Family Member 3 | 300265 |
| **CHD Risk Genes - Mouse** | **OMIM** |
| *Acan*, Aggrecan | 155760 |
| *Adamts6*, Adam metalloprotease with thrombospondin type 1 motif 6 | 605008 |
| *Anks6*, Ankyrin repeat and sterile alpha motif domain containing 6 | 615370 |
| *Ap1b1*, Adaptor related protein complex 1 beta 1 subunit | 600157 |
| *Ap2b1*, Adaptor related protein complex 2 beta 1 subunit | 601025 |
| *Bicc1*, BicC family RNA binding protein 1 | 614295 |
| *Cc2d2a*, Coiled-coil and C2 domain containing 2A | 612013 |
| *Cntrl*, Centriolin | 605496 |
| *NAT8*, N-Acetyltransferase 8 (putative) | 606716 |
| *CXCR4*, C-X-C motif chemokine receptor 4 | 162643 |
| *DAW1*, Dynein assembly factor with WD repeats 1 | NA |
| *DCTN5*, Dynactin 5 | 612962 |
| *DNM2*, Dynamin 2 | 602378 |
| *DRC1*, Dynein regulatory complex subunit 1 | 615288 |
| *FOXJ1*, Forkhead box J1 | 602291 |
| *FREM2*, FRAS1 related extracellular matrix protein 2 | 608945 |
| *FUZ*, Fuzzy planar cell polarity protein | 610622 |
| *C1ORF127*, Chromosome 1 open reading frame 127 | NA |
| *Hectd1*, HECT domain E3 ubiquitin protein ligase 1 | NA |
| *Ift74*, Intraflaggelar transport 74 | 608040 |
| *Lox*, Lysyl oxidase | 153455 |
| *Lrp1*, LDL receptor related protein 1 | 107770 |
| *Ltbp1*, Latent TGF-beta binding protein 1 | 150390 |
| *Mmp21*, Matrix metalloprotease 21 | 608416 |
| *Myh10*, Myosin heavy chain 10 | 160776 |
| *Ndst1*, N-Deacetylase and N-sulfotransferase 1 | 600853 |
| *Pcsk5*, Proprotein convertase subtilisin/kexin type 5 | 600488 |
| *Pde2a*, Phosphodiesterase 2A | 602658 |
| *Plxnd1*, Plexin D1 | 604282 |
| *Prdm1*, PR/SET domain 1 | 603423 |
| *Prickle1*, Prickle planar cell polarity protein 1 | 608500 |
| *Pskh1*, Protein serine kinase H1 | 177015 |
| *Ptk7*, Protein tyrosine kinase 7 (inactive) | 601890 |
| *Robo1*, Roundabout guidance receptor 1 | 602430 |
| *Smad6*, SMAD family member 6 | 602931 |
| *Snx17*, Sorting nexin 17 | 605963 |
| *Sufu*, Suppressor of fused | 607035 |
| *Tab1*, TAK1 binding protein | 602615 |
| *Tbc1d32*, TBC1 domain family member 32 | 615867 |
| *Tmem67*, Transmembrane protein 67 | 609884 |
| *Zbtb14*, Zinc finger and BTB domain containing 14 | 602126 |

**Table S2. Rare, predicted deleterious variants in known PAH risk genes identified in 258 PAH-CHD patients. Patients are heterozygous for the indicated variants.**

| **ID** | **Gender** | **Age, dx or enrollment (y)** | **Associated medical conditions^a^** | **Gene** | **Transcript** | **Exon** | **Nucleotide change** | **AA change** | **Inheritance** | **Previously-reported?** | **Allele frequency (ExAC)^b^** | **CADD** | **REVEL ^c^** |
| --- | --- | --- | --- | --- | --- | --- | --- | --- | --- | --- | --- | --- | --- |
| JM188 | F | 17 | ASD, cardiomegaly, developmental delays | *BMPR1A* | NM_004329 | 13 | c.A1498G | p.M500V | maternal | rs376651641 | 4.94E-05 | 25.2 | 0.63 |
| JM177 | F | 10 | ASD | *BMPR1B* | NM_001256792 | 6 | c.T547C | p.S183P | maternal | No | 2.49E-05 | 25 | 0.751 |
| FPPH079 | F | 43 | ASD, PFO | *BMPR2* | NM_001204 | 9 | c.1276+1G>A |  | unknown | Machado et.al. 2009 rs767070218 | --- | . | . |
| FPPH112 | F | 16 | ASD | *BMPR2* | NM_001204 | 9 | c.1271_1277 delinsAGA^d^ | p.F424X | unknown | Machado et.al. 2009 | --- | . | . |
| FPPH119 | F | 1 | ASD | *BMPR2* | NM_001204 | 4 | c.419-43 del |  | maternal | Machado et.al. 2009 | --- | . | . |
| JM0001 | M | 16 | VSD | *BMPR2* | NM_001204 | 4 | c.529+1G>A | . | unknown | Machado et.al. 2009 | --- | 27.5 | . |
| JM150 | M | 15 | ASD, PDA, VSD | *BMPR2* | NM_001204 | 11 | c.A1509C | p.E503D | unknown | Machado et.al. 2009 | --- | 18.28 | 0.662 |
| JM765 | M | 3 | TGV, tachycardia | *BMPR2* | NM_001204 | 12 | c.G2353A | p.E785K | maternal | No | 8.24E-06 | 32 | 0.587 |
| JM887 | F | 4 | VSD, PDA | *BMPR2* | NM_001204 | 2 | c.G211A | p.E71K | unknown | No | --- | 26.4 | 0.751 |
| JM226 | F | 38 | VSD | *CAV1* | NM_001172896 | 2 | c.A145G | p.S49G | unknown | No | --- | 24.8 | 0.892 |
| FPPH104-01 | F | 38 | ASD, HHT | *ENG* | NM_000118.3 | 12 | c.1633G>A | p.G545S | maternal | rs142896669 | --- | . | . |
| JM1365 | F | newborn | ASD, CoA, great vein anomaly, hypothyroidism | *SMAD9* | NM_001127217 | 2 | c.C204A | p.C68X | unknown | No | --- | 32 | . |
| JM147 | F | 15 | ASD | *SMAD9* | NM_005905 | 5 | c.A1045G | p.N349D | unknown | No | 3.30E-05 | 24.2 | 0.536 |
| JM0002 | F | 2 | ASD, VSD | *TBX4* | NM_018488 | 3 | c.C293G | p.P98R | *de novo* | No | --- | 31 | 0.973 |
| JM0060 | M | 11 | VSD, Eisenmenger syndrome | *TBX4* | NM_018488 | 8 | c.1070delC | p.A357fs | paternal or *de novo* | No | --- | 39 | . |
| JM1148 | F | <1 | ASD, PFO | *TBX4* | NM_018488 | 5 | c.702+1G>A | . | unknown | No | --- | 27.3 | . |
| JM1426 | M | <1 | ASD, PDA, sleep apnea | *TBX4* | NM_018488 | 8 | c.1106delC | p.S369fs | maternal | No | --- | 39 | . |
| JM847 | M | newborn | alveolar hypoplasia, PFO | *TBX4* | NM_018488 | 4 | c.537_546del | p.D179fs | *de novo* | No | --- | 39 | . |
| JM950 | F | <1 | VSD | *TBX4* | NM_018488 | 6 | c.G749A | p.R250Q | unknown | No | --- | 34 | 0.947 |
| JM889 | F | 5 | PDA, PFO | *TBX4* | NM_018496 | 5 | c.670_672delTTC | p.223_224del | *de novo* | No | --- | . | . |

^a^ASD, atrial septal defect; CoA, coartation of the artery; HHT, hereditary hemorrhagic telangiectasia; PDA, patent ductus arteriosus; PFO, patent foramen ovalae; TGV, transposition of the great vessels; VSD, ventricular septal defect.

^b^---, mutation not present in ExAC.

^c^Deleterious variants defined by ExAC and gnomAD allele frequency < 0.01% and REVEL >0.5.

^d^Variant spans an intron and was not verified by direct sequencing of cDNA. Designated as NC_000002.11:g.203397450_203407034delinsAGA in ClinVar.

**Table S3a. Similar frequency of rare synonymous variants among European cases and non-Finnish**

**European gnomAD individuals.**

| **Mutation type^a^** | **PAH-CHD cases (n=143)** | **gnomAD WGS controls (n=7509)** | **Enrichment** | **p-value** |
| --- | --- | --- | --- | --- |
|  |  |  |  |  |
| SYN | 3,421 | 176,010 | 1.01 | 0.434 |
| LGD | 574 | 27,312 | 1.10 | 0.031 |
| MIS | 7,335 | 369,216 | 1.04 | 0.003 |

^a^SYN, synonymous; LGD, likely gene damaging; MIS, missense.

**Table S3b. Enrichment of rare, predicted deleterious variants in PAH but not CHD risk**

**genes in European PAH-CHD cases.**

| **Gene set** | **Variant class^a^** | **PAH-CHD cases (n=143)** | **gnomAD WGS controls (n=7,509)** | **Enrichment** | **p-value** |
| --- | --- | --- | --- | --- | --- |
| **PAH (11)** | SYN | 2 | 83 | 1.26 | 0.676 |
|  | LGD | 2 | 15 | **6.95** | **0.040** |
|  | D-Mis | 4 | 40 | **5.21** | **0.009** |
|  | D-Mis+LGD | 6 | 55 | **5.69** | **0.001** |
| **CHD (253)** | SYN | 87 | 4,799 | 0.95 | 0.67 |
|  | LGD | 11 | 516 | 1.11 | 0.63 |
|  | D-Mis | 44 | 2,131 | 1.08 | 0.58 |
|  | D-Mis+LGD | 55 | 2,647 | 1.08 | 0.52 |

^a^SYN, synonymous; LGD, likely gene damaging; D-Mis, damaging missense predicted by REVEL >0.5.

**Table S5. Enrichment of rare, predicted deleterious variants in 1,947 SOX17 target genes.**

| **Mutation type**^a^ | **Observed in cases (n=143)** | **Observed in controls** | **Enrichment** | **p-value** |
| --- | --- | --- | --- | --- |
|  |  | **(n=7509)** |  |  |
| SYN | 263 | 13,615 | 1.01 | 0.901 |
| LGD | 32 | 1,771 | 0.94 | 0.862 |
| MIS | 618 | 27,771 | **1.16** | **3.39E-04** |
| D-Mis | 131 | 5,525 | **1.24** | **0.019** |

^a^SYN, synonymous; LGD, likely gene damaging; MIS, missense; D-Mis, damaging missense predicted by REVEL >0.5

**Table S6.** **SOX17 target gene expression rank in murine 14.5E developing heart^a^ and human adult pulmonary artery endothelial cells (PAEC)^b^.** The list includes 149 genes with PAH-CHD patient-derived rare deleterious variants.

| **Gene symbol** | **Chrom** | **Nucleotide change** | **Amino acid change** | **Variant type** | **Allele frequency (ExAC)** | **CADD** | **REVEL** | **Heart rank** | **PAEC rank** |
| --- | --- | --- | --- | --- | --- | --- | --- | --- | --- |
| *SLC8A1* | 2 | c.C2782T | p.R928W | Dmis | 2.47E-05 | 34 | 0.584 | 99.67 | 30.85 |
| *SLC8A1* | 2 | c.C2531T | p.A844V | Dmis | 0 | 29 | 0.66 | 99.67 | 30.85 |
| *VIM* | 10 | c.C797T | p.T266M | Dmis | 2.48E-05 | 34 | 0.719 | 99.62 | 99.93 |
| *PTMA* | 2 | c.261_262insT | p.T87fs | frameshift | 0 | 35 | . | 99.49 | 99.47 |
| *LAMC1* | 1 | c.G230A | p.C77Y | Dmis | 0 | 27 | 0.775 | 99.22 | 97.35 |
| *RYR2* | 1 | c.G1864A | p.G622R | Dmis | 1.46E-05 | 32 | 0.803 | 98.53 | 0.00 |
| *RYR2* | 1 | c.C5056T | p.L1686F | Dmis | 3.32E-05 | 21.7 | 0.54 | 98.53 | 0.00 |
| *RYR2* | 1 | c.A6212C | p.Q2071P | Dmis | 0 | 27.1 | 0.841 | 98.53 | 0.00 |
| *HBP1* | 7 | c.A1464T | p.E488D | Dmis | 0 | 26.3 | 0.619 | 98.44 | 90.51 |
| *WNK1* | 12 | c.C1088T | p.S363L | Dmis | 0 | 25.3 | 0.599 | 98.13 | 84.78 |
| *WNK1* | 12 | c.G5830T | p.D1944Y | Dmis | 1.65E-05 | 29.2 | 0.6 | 98.13 | 84.78 |
| *SOD1* | 21 | c.C346T | p.R116C | Dmis | 0 | 35 | 0.98 | 97.21 | 95.68 |
| *MCAM* | 11 | c.T1541C | p.L514P | Dmis | 0 | 31 | 0.504 | 97.06 | 99.60 |
| *LAMA4* | 6 | c.429delA | p.A143fs | frameshift | 0 | 35 | . | 96.97 | 98.03 |
| *NF1* | 17 | c.C7027T | p.R2343W | Dmis | 0 | 35 | 0.551 | 96.96 | 92.68 |
| *MAT2A* | 2 | c.A44C | p.E15A | Dmis | 0 | 22.8 | 0.508 | 96.76 | 94.64 |
| *PPL* | 16 | c.2358delG | p.K786fs | frameshift | 0 | 25.1 | . | 96.43 | 89.11 |
| *EGF* | 4 | c.G2033A | p.R678H | Dmis | 0 | 27.5 | 0.695 | 95.99 | 97.72 |
| *EGF* | 4 | c.G2840T | p.G947V | Dmis | 0 | 26 | 0.847 | 95.99 | 97.72 |
| *EGF* | 4 | c.G2846T | p.C949F | Dmis | 0 | 27.6 | 0.94 | 95.99 | 97.72 |
| *EGF* | 4 | c.C1063T | p.Q355X | stop gain | 1.65E-05 | 34 | . | 95.99 | 97.72 |
| *CNN3* | 1 | c.T545G | p.I182S | Dmis | 0 | 28 | 0.638 | 95.59 | 97.83 |
| *BZW2* | 7 | c.G1209C | p.E403D | Dmis | 0 | 24.2 | 0.548 | 95.56 | 85.45 |
| *BZW2* | 7 | c.C37T | p.R13W | Dmis | 1.65E-05 | 25.3 | 0.72 | 95.56 | 85.45 |
| *BZW2* | 7 | c.G1191C | p.Q397H | Dmis | 0 | 32 | 0.696 | 95.56 | 85.45 |
| *RPS25* | 11 | c.A86G | p.K29R | Dmis | 0 | 24.9 | 0.544 | 95.53 | 98.59 |
| *TOP1* | 20 | c.C773T | p.T258M | Dmis | 0 | 32 | 0.561 | 95.42 | 63.42 |
| *BAZ1B* | 7 | c.2660delT | p.F887fs | frameshift | 0 | 35 | . | 95.29 | 83.35 |
| *BAZ1B* | 7 | c.A1949G | p.Y650C | Dmis | 0 | 25 | 0.532 | 95.29 | 83.35 |
| *ANK3* | 10 | c.2614+1G>T |  | splicing | 0 | 28.2 | . | 95.01 | 77.93 |
| *ANK3* | 10 | c.A976T | p.I326F | Dmis | 0 | 23.7 | 0.571 | 95.01 | 77.93 |
| *EPHB4* | 7 | c.C2324T | p.T775M | Dmis | 2.47E-05 | 30 | 0.7 | 94.28 | 96.08 |
| *TCF4* | 18 | c.G811T | p.G271C | Dmis | 8.28E-06 | 35 | 0.543 | 93.63 | 96.46 |
| *ASPH* | 8 | c.A1553G | p.Y518C | Dmis | 8.26E-06 | 29.6 | 0.749 | 93.57 | 92.02 |
| *GAB1* | 4 | c.C274T | p.R92W | Dmis | 0 | 34 | 0.691 | 90.63 | 47.95 |
| *ETFDH* | 4 | c.T1631C | p.V544A | Dmis | 8.24E-06 | 19.5 | 0.554 | 90.62 | 52.35 |
| *PTPRK* | 6 | c.G693T | p.E231D | Dmis | 8.25E-06 | 27.9 | 0.645 | 90.50 | 78.97 |
| *PSMC4* | 19 | c.A122G | p.Y41C | Dmis | 0 | 27.5 | 0.88 | 90.49 | 92.58 |
| *C9* | 5 | c.1073dupT | p.L358fs | frameshift | 4.13E-05 | 35 | . | 90.27 | 84.32 |
| *C9* | 5 | c.T902C | p.I301T | Dmis | 0 | 24.4 | 0.584 | 90.27 | 84.32 |
| *SLC2A4* | 17 | c.C1054T | p.L352F | Dmis | 0 | 27.2 | 0.788 | 90.24 | 45.06 |
| *USP5* | 12 | c.A536C | p.H179P | Dmis | 0 | 26.9 | 0.739 | 90.08 | 90.75 |
| *USP5* | 12 | c.C2083T | p.H695Y | Dmis | 1.71E-05 | 33 | 0.618 | 90.08 | 90.75 |
| *MEIS1* | 2 | c.C699G | p.S233R | Dmis | 0 | 19.21 | 0.588 | 89.64 | 64.47 |
| *LEPR* | 1 | c.G1874A | p.W625X | stop gain | 0 | 39 | . | 89.27 | 74.94 |
| *PRKCSH* | 19 | c.A1179C | p.Q393H | Dmis | 0 | 24.3 | 0.53 | 88.97 | 93.66 |
| *EP300* | 22 | c.G5307C | p.K1769N | Dmis | 0 | 25 | 0.524 | 88.15 | 67.63 |
| *PHLDB2* | 3 | c.G3191C | p.R1064P | Dmis | 0 | 34 | 0.673 | 87.94 | 84.87 |
| *SEC23IP* | 10 | c.T2843C | p.I948T | Dmis | 0 | 25.7 | 0.587 | 87.30 | 60.74 |
| *PRICKLE1* | 12 | c.C788T | p.A263V | Dmis | 0 | 27.2 | 0.623 | 86.60 | 73.72 |
| *MYEF2* | 15 | c.C1363T | p.R455W | Dmis | 0 | 35 | 0.524 | 86.45 | 65.40 |
| *PTPN14* | 1 | c.A916G | p.K306E | Dmis | 0 | 25.3 | 0.556 | 85.45 | 76.88 |
| *PHF12* | 17 | c.C1228G | p.P410A | Dmis | 1.65E-05 | 21.7 | 0.608 | 85.26 | 40.73 |
| *CCDC50* | 3 | c.C131A | p.S44X | stop gain | 4.12E-05 | 44 | . | 84.98 | 90.61 |
| *SMARCA2* | 9 | c.G182C | p.G61A | Dmis | 9.63E-06 | 23.7 | 0.511 | 84.17 | 76.00 |
| *LZTR1* | 22 | c.G2131A | p.G711R | Dmis | 5.79E-05 | 34 | 0.642 | 84.12 | 88.51 |
| *PHYH* | 10 | c.T9A | p.D3E | Dmis | 0 | 22.5 | 0.624 | 84.12 | 34.48 |
| *FTSJ3* | 17 | c.T1208C | p.L403P | Dmis | 0 | 27.6 | 0.604 | 83.76 | 87.43 |
| *FTSJ3* | 17 | c.T578C | p.I193T | Dmis | 8.24E-06 | 26.4 | 0.543 | 83.76 | 87.43 |
| *MME* | 3 | c.C425T | p.S142F | Dmis | 0 | 34 | 0.63 | 83.25 | 54.72 |
| *LLGL1* | 17 | c.G1096A | p.E366K | Dmis | 0 | 33 | 0.546 | 82.38 | 66.02 |
| *POLG* | 15 | c.C2585T | p.A862V | Dmis | 0 | 33 | 0.968 | 81.27 | 87.78 |
| *POLG* | 15 | c.C1270G | p.L424V | Dmis | 0 | 24.9 | 0.696 | 81.27 | 87.78 |
| *POLG* | 15 | c.G926A | p.R309H | Dmis | 2.50E-05 | 35 | 0.955 | 81.27 | 87.78 |
| *SEC24B* | 4 | c.A3169G | p.S1057G | Dmis | 0 | 25.8 | 0.687 | 80.84 | 45.21 |
| *SEC24B* | 4 | c.T3532C | p.F1178L | Dmis | 2.49E-05 | 26 | 0.587 | 80.84 | 45.21 |
| *ENC1* | 5 | c.C106T | p.R36W | Dmis | 1.65E-05 | 32 | 0.561 | 80.57 | 87.33 |
| *PANK1* | 10 | c.A443G | p.Y148C | Dmis | 0 | 26 | 0.849 | 80.11 | 41.67 |
| *CAPN7* | 3 | c.A1C | p.M1L | Dmis | 0 | 22.8 | 0.756 | 79.24 | 61.98 |
| *CAPN7* | 3 | c.C1738T | p.Q580X | stop gain | 0 | 40 | . | 79.24 | 61.98 |
| *SNX6* | 14 | c.A572C | p.K191T | Dmis | 8.84E-06 | 29.3 | 0.612 | 78.54 | 90.09 |
| *ETV1* | 7 | c.A1061G | p.Y354C | Dmis | 0 | 24.9 | 0.939 | 77.95 | 34.92 |
| *SIRT2* | 19 | c.C272T | p.P91L | Dmis | 1.65E-05 | 34 | 0.673 | 77.73 | 73.68 |
| *THBS1* | 15 | c.C2428T | p.R810W | Dmis | 2.47E-05 | 29.2 | 0.726 | 77.02 | 99.98 |
| *MITF* | 3 | c.C710T* | p.P237L | Dmis | 0 | 23.7 | 0.833 | 76.93 | 26.16 |
| *LIFR* | 5 | c.1107_1113del | p.Y369fs | frameshift | 0 | 33 | . | 76.81 | 67.48 |
| *CASD1* | 7 | c.T2073A | p.Y691X | stop gain | 8.27E-06 | 38 | . | 76.81 | 48.04 |
| *EPC1* | 10 | c.G1214T | p.R405L | Dmis | 3.30E-05 | 34 | 0.53 | 76.78 | 50.06 |
| *DHX38* | 16 | c.C880T | p.R294X | stop gain | 0 | 36 | . | 76.12 | 90.11 |
| *NSMAF* | 8 | c.G818A | p.R273H | Dmis | 1.65E-05 | 24 | 0.678 | 75.78 | 72.19 |
| *MSH2* | 2 | c.A2362C | p.T788P | Dmis | 8.24E-06 | 28.7 | 0.915 | 75.56 | 68.65 |
| *ATE1* | 10 | c.T770C | p.V257A | Dmis | 1.72E-05 | 28.5 | 0.852 | 75.27 | 67.15 |
| *SAMHD1* | 20 | c.G677T | p.R226L | Dmis | 0 | 29.7 | 0.567 | 74.08 | 60.51 |
| *ARHGAP10* | 4 | c.C2041T | p.R681X | stop gain | 8.24E-06 | 45 | . | 73.72 | 60.66 |
| *TBC1D10B* | 16 | c.C2126T | p.T709I | Dmis | 0 | 26.1 | 0.53 | 73.35 | 70.83 |
| *THYN1* | 11 | Tc.C354G | p.C118W | Dmis | 0 | 29.6 | 0.726 | 72.97 | 84.20 |
| *MED6* | 14 | c.C244T | p.R82W | Dmis | 3.32E-05 | 34 | 0.872 | 71.70 | 63.48 |
| *REV3L* | 6 | c.A185G | p.Y62C | Dmis | 4.28E-05 | 26.2 | 0.645 | 71.62 | 51.95 |
| *RBM28* | 7 | c.G1199C | p.G400A | Dmis | 2.49E-05 | 31 | 0.584 | 70.76 | 68.91 |
| *NDRG3* | 20 | c.G82A* | p.G28S | Dmis | 0 | 32 | 0.558 | 70.67 | 53.43 |
| *SMARCAL1* | 2 | c.T1317G | p.F439L | Dmis | 4.94E-05 | 27.6 | 0.722 | 69.25 | 43.26 |
| *PPIL4* | 6 | c.A908G | p.N303S | Dmis | 0 | 26 | 0.588 | 69.21 | 60.01 |
| *MKKS* | 20 | c.T1082C | p.F361S | Dmis | 0 | 25.7 | 0.507 | 68.63 | 32.45 |
| *PNPLA6* | 19 | c.3360delC | p.H1120fs | frameshift | 8.41E-06 | 35 | . | 68.51 | 87.72 |
| *OPA3* | 19 | c.G400T | p.E134X | stop gain | 0 | 37 | . | 66.82 | 37.64 |
| *WRN* | 8 | c.524delG | p.W175X | stop gain | 8.24E-06 | . | . | 66.37 | 86.56 |
| *PGS1* | 17 | c.G734A | p.R245H | Dmis | 8.29E-06 | 34 | 0.687 | 66.06 | 68.99 |
| *VANGL1* | 1 | c.T541G | p.F181V | Dmis | 0 | 22.6 | 0.545 | 65.74 | 45.15 |
| *VANGL1* | 1 | c.C970T | p.R324W | Dmis | 8.25E-06 | 34 | 0.846 | 65.74 | 45.15 |
| *VANGL1* | 1 | c.C970T | p.R324W | Dmis | 8.25E-06 | 34 | 0.846 | 65.74 | 45.15 |
| *TMEM70* | 8 | c.A497G | p.Y166C | Dmis | 0 | 26.9 | 0.906 | 63.59 | 60.72 |
| *KIF21A* | 12 | c.2267delC | p.T756fs | frameshift | 0 | 35 | . | 63.55 | 47.94 |
| *ITPR3* | 6 | c.2889delG | p.V963fs | frameshift | 8.25E-06 | 35 | . | 63.13 | 79.28 |
| *ITPR3* | 6 | c.A309T | p.Q103H | Dmis | 3.36E-05 | 17.97 | 0.781 | 63.13 | 79.28 |
| *ITPR3* | 6 | c.T2208G | p.F736L | Dmis | 0 | 23.6 | 0.66 | 63.13 | 79.28 |
| *ITPR3* | 6 | c.C3583T | p.R1195W | Dmis | 8.27E-06 | 34 | 0.748 | 63.13 | 79.28 |
| *ITPR3* | 6 | c.G5038A | p.G1680R | Dmis | 0 | 24 | 0.629 | 63.13 | 79.28 |
| *SPRED2* | 2 | c.T119C | p.V40A | Dmis | 0 | 23.4 | 0.919 | 62.07 | 21.08 |
| *ALS2* | 2 | c.C2864T | p.P955L | Dmis | 0 | 26.1 | 0.512 | 62.03 | 70.57 |
| *ALS2* | 2 | c.C2061G | p.N687K | Dmis | 0 | 23.6 | 0.592 | 62.03 | 70.57 |
| *TBC1D24* | 16 | c.T1609C | p.F537L | Dmis | 0 | 25.4 | 0.681 | 61.57 | 43.44 |
| *PRDM1* | 6 | c.A80G | p.Y27C | Dmis | 0 | 26 | 0.91 | 60.75 | 36.19 |
| *EZH1* | 17 | c.C811A | p.P271T | Dmis | 1.65E-05 | 24.6 | 0.689 | 60.68 | 43.59 |
| *PITX2* | 4 | c.G970C | p.V324L | Dmis | 1.67E-05 | 31 | 0.66 | 57.50 | 0.00 |
| *GPSM2* | 1 | c.G316T | p.G106C | Dmis | 2.57E-05 | 34 | 0.81 | 57.30 | 58.40 |
| *COL27A1* | 9 | c.T708G | p.C236W | Dmis | 8.26E-06 | 22.5 | 0.566 | 55.03 | 35.24 |
| *ABCC1* | 16 | c.A830C | p.Y277S | Dmis | 0 | 21.1 | 0.557 | 55.03 | 75.10 |
| *ALG3* | 3 | c.A1G | p.M1V | Dmis | 0 | 25.3 | 0.637 | 54.03 | 57.83 |
| *SLC1A3* | 5 | c.C1001T | p.A334V | Dmis | 0 | 35 | 0.577 | 53.23 | 4.25 |
| *MYH2* | 17 | c.A3931C | p.K1311Q | Dmis | 0 | 27.5 | 0.831 | 53.02 | 0.00 |
| *MYH2* | 17 | c.A2669T | p.K890I | Dmis | 0 | 28.4 | 0.898 | 53.02 | 0.00 |
| *MYH2* | 17 | c.C2014T | p.H672Y | Dmis | 0 | 24.6 | 0.738 | 53.02 | 0.00 |
| *STARD13* | 13 | c.G1058A | p.G353D | Dmis | 2.47E-05 | 29.1 | 0.525 | 52.96 | 63.89 |
| *SMYD4* | 17 | c.C1586A | p.T529K | Dmis | 2.35E-05 | 32 | 0.721 | 49.70 | 41.82 |
| *UPP1* | 7 | c.C344A | p.S115X | stop gain | 0 | 37 | . | 49.17 | 76.37 |
| *TSEN2* | 3 | c.T841G | p.L281V | Dmis | 0 | 24.6 | 0.844 | 48.74 | 45.48 |
| *TSEN2* | 3 | c.T1022C | p.L341P | Dmis | 8.27E-06 | 28.1 | 0.606 | 48.74 | 45.48 |
| *WDR34* | 9 | c.A347T | p.E116V | Dmis | 0 | 23.5 | 0.656 | 48.19 | 87.40 |
| *TMEM104* | 17 | c.G1337A | p.R446H | Dmis | 0 | 33 | 0.639 | 47.15 | 34.66 |
| *ATG10* | 5 | c.C494T | p.P165L | Dmis | 4.12E-05 | 33 | 0.828 | 46.72 | 86.41 |
| *DIMT1* | 5 | c.C484T | p.R162X | stop gain | 1.65E-05 | 36 | . | 46.23 | 71.61 |
| *OSGEPL1* | 2 | c.A133T | p.S45C | Dmis | 8.30E-06 | 28 | 0.776 | 46.13 | 32.92 |
| *SH3TC2* | 5 | c.C335G | p.T112S | Dmis | 8.24E-06 | 23.7 | 0.529 | 43.41 | 22.74 |
| *FUT10* | 8 | c.G721A | p.D241N | Dmis | 8.24E-06 | 26.5 | 0.509 | 43.09 | 55.23 |
| *EMB* | 5 | c.142_143del | p.E48fs | frameshift | 0 | 25.3 | . | 41.21 | 0.00 |
| *TEAD4* | 12 | c.C175A | p.P59T | Dmis | 0 | 27.8 | 0.615 | 40.66 | 61.00 |
| *CLCN5* | X | c.T1892C | p.L631P | Dmis | 0 | 25.1 | 0.934 | 40.07 | 42.28 |
| *BBS5* | 2 | c.G413A | p.R138H | Dmis | 0 | 33 | 0.828 | 39.99 | 25.70 |
| *LAMA1* | 18 | c.A7084G | p.R2362G | Dmis | 0 | 24.3 | 0.529 | 39.87 | 4.84 |
| *SOX17* | 8 | c.344delG | p.R115fs | frameshift | 0 | 35 | . | 38.86 | 84.29 |
| *SOX17* | 8 | c.489_510del | p.Q163fs | frameshift | 0 | 33 | . | 38.86 | 84.29 |
| *SOX17* | 8 | c.489_510del | p.Q163fs | frameshift | 0 | 33 | . | 38.86 | 84.29 |
| *SOX17* | 8 | c.A226G | p.M76V | Dmis | 0 | 26.3 | 0.967 | 38.86 | 84.29 |
| *SOX17* | 8 | c.C388T | p.Q130X | stop gain | 0 | 39 | . | 38.86 | 84.29 |
| *SLC16A13* | 17 | c.C498A | p.Y166X | stop gain | 0 | 35 | . | 38.76 | 20.53 |
| *NUP35* | 2 | c.A644G | p.Y215C | Dmis | 0 | 27.2 | 0.714 | 38.23 | 57.36 |
| *FRZB* | 2 | c.G826T | p.E276X | stop gain | 0 | 45 | . | 36.95 | 2.51 |
| *ABCC4* | 13 | c.G3518A | p.R1220Q | Dmis | 5.77E-05 | 35 | 0.607 | 36.86 | 68.06 |
| *GLIPR1* | 12 | c.G351A | p.W117X | stop gain | 8.24E-06 | 37 | . | 36.30 | 86.81 |
| *ATP7B* | 13 | c.T2942C | p.L981P | Dmis | 0 | 27.7 | 0.936 | 35.90 | 32.47 |
| *ROBO2* | 3 | c.C1816A | p.P606T | Dmis | 0 | 27.5 | 0.694 | 34.79 | 13.96 |
| *KLB* | 4 | c.2947_2948del | p.T983fs | frameshift | 0 | 26.3 | . | 32.55 | 90.58 |
| *THRSP* | 11 | c.T221C | p.L74P | Dmis | 0 | 27.2 | 0.601 | 27.60 | 0.00 |
| *GIPC2* | 1 | c.T131C | p.F44S | Dmis | 5.51E-05 | 33 | 0.906 | 27.02 | 58.27 |
| *NR5A2* | 1 | c.C625T | p.P209S | Dmis | 0 | 21.2 | 0.532 | 25.70 | 53.54 |
| *LIPG* | 18 | c.G713C | p.R238P | Dmis | 0 | 34 | 0.73 | 25.35 | 77.78 |
| *SELP* | 1 | c.C950T | p.P317L | Dmis | 0 | 28.7 | 0.635 | 23.17 | 21.84 |
| *LIPH* | 3 | c.G469C | p.A157P | Dmis | 0 | 32 | 0.969 | 21.34 | 1.91 |
| *CCT6B* | 17 | c.G509A | p.G170D | Dmis | 0 | 32 | 0.632 | 19.22 | 7.37 |
| *PCOLCE2* | 3 | c.G1058A | p.G353D | Dmis | 4.12E-05 | 27.8 | 0.684 | 18.88 | 35.56 |
| *SLC4A9* | 5 | c.C2042A | p.T681K | Dmis | 0 | 29.7 | 0.721 | 16.37 | 0.00 |
| *TM4SF20* | 2 | c.184-2A>T |  | splicing | 2.47E-05 | 23.5 | . | 15.57 | 0.00 |
| *A4GNT* | 3 | c.A500G | p.D167G | Dmis | 3.30E-05 | 27.2 | 0.973 | 13.88 | 0.00 |
| *MFI2* | 3 | c.A1681G | p.R561G | Dmis | 0 | 27.6 | 0.683 | 10.22 | NA |
| *CHST9* | 18 | c.G166T | p.G56X | stop gain | 1.71E-05 | 35 | . | 0.00 | 0.00 |

^a^Data reported in Zaidi et al. 2013. Nature. 498: 220-223.

^a^ENCODE RNA-seq data, ENCBS024RNA

**Table S7. Biological pathway analysis of SOX17 target genes harboring PAH-CHD patient-derived rare deleterious variants.** Data were obtained using Reactome 2016 through Enrichr (amp/pharma.mssm.edu/Enrichr/enrich). All pathways with FDR-adjusted p-value ≤0.05 are listed.

| **Term** | **Reactome ID** | **#Genes in overlap** | **P-value** | **Adjusted P-value** | **Genes** |
| --- | --- | --- | --- | --- | --- |
| Developmental biology | R-HAS-1266738 | 16/786 | 6.82E-05 | 0.03 | *KLB*, *ROBO2*, *LAMA1*, *EGF*, *ANK3*, *LAMC1*, *SLC2A4*, *MED6*, *SPRED2*, *MEIS1*, *NR5A2*, *PSMC4*, *NF1*, *EP300*, *TCF4*, *EPHB4* |
| Transmembrane transport of small molecules | R-HAS-382561 | 13/594 | 1.69E-04 | 0.03 | *RYR2*, *ABCC4*, *ABCC1*, *SLC1A3*, *SLC2A4*, *SLC8A1*, *CLCN5*, *SLCA9*, *ATP7B*, *ASPH*, *WNK1*, *NUP35*, *EMB* |
| Non-integrin membrane ECM interactions | R-HAS-3000171 | 4/42 | 1.72E-04 | 0.03 | *LAMA1*, *LAMA4*, *LAMC1*, *THBS1* |
| Ion homeostasis | R-HAS-5578775 | 4/51 | 3.67E-04 | 0.05 | *RYR2*, A*SPH*, *TPR3*, *SLC8A1* |
| Laminin interactions | R-HAS-3000157 | 3/23 | 4.62E-04 | 0.05 | *LAMA1*, *LAMA4*, *LAMC1* |

**Table S8. List of all rare *de novo* variants in pediatric-onset PAH-CHD trios (n=60).**

| **Proband ID** | **Variant type** | **Gene** | **Transcript** | **Exon** | **Nucleotide Change** | **AA Change** | **pLI** | **LOF_z** | **Mis_z** | **Allele frequency (ExAC)** |
| --- | --- | --- | --- | --- | --- | --- | --- | --- | --- | --- |
| JM0002 | Missense | *TBX4* | NM_018488 | 3 | c.C293G | p.P98R | 0.41 | 3.26 | 1.57 | 0 |
| JM847 | Frameshift | *TBX4* | NM_018488 | 4 | c.536_546A | p.D179fs | 0.41 | 3.26 | 1.57 | 0 |
| JM889 | Inframe | *TBX4* | NM_018488 | 5 | c.668_671C | p.223_224del | 0.41 | 3.26 | 1.57 | 0 |
| JM140 | Missense | *BRWD3* | NM_153252 | 12 | c.G1087T | p.D363Y | 1 | 7.21 | 4.18 | 0 |
| JM1357 | Missense | *AFF2* | NM_001170628 | 8 | c.C974G | p.P325R | 1 | 4.87 | 0.83 | 0 |
| JM839 | Missense | *L1CAM* | NM_001143963 | 26 | c.A3661G | p.S1221G | 1 | 5.71 | 2.91 | 0 |
| JM217 | Missense | *FOXD3* | NM_012183 | 1 | c.G319T | p.G107C | NA | NA | NA | 0 |
| JM217 | Missense | *DNMT3A* | NM_001320893 | 4 | c.T584C | p.L195P | 0 | -3.54 | 4.31 | 0 |
| JM696 | Missense | *C2orf71* | NM_001029883 | 2 | c.G3803A | p.R1268Q | 0 | 1.03 | -3.8 | 2.22E-05 |
| JM214 | Missense | *EXOC6B* | NM_001321734 | 10 | c.C827T | p.S276L | 0.67 | 4.59 | 1.44 | 0 |
| JM176 | Missense | *LOXL3* | NM_001289165 | 3 | c.G17T | p.G6V | 0 | 2.58 | 2.01 | 0 |
| JM237 | Missense | *IMMT* | NM_001100169 | 5 | c.C485T | p.A162V | 0 | 3.21 | -0.47 | 0 |
| JM138 | Missense | *RASA2* | NM_001303245 | 19 | c.C1916T | p.T639I | 0 | 1.33 | 1.32 | 2.65E-05 |
| JM0043 | Missense | *FAM149A* | NM_001006655 | 12 | c.C1290G | p.F430L | 0.01 | 2.68 | -0.67 | 0 |
| JM892 | Missense | *DNAH5* | NM_001369 | 6 | c.C746T | p.T249I | 0 | 6.01 | -1.75 | 0 |
| JM892 | Missense | *CMYA5* | NM_153610 | 2 | c.G2653A | p.E885K | 0 | 0.2 | -2.73 | 1.67E-05 |
| JM187 | Missense | *HIST1H1C* | NM_005319 | 1 | c.C482T | p.P161L | NA | NA | NA | 8.27E-06 |
| JM198 | Missense | *C7orf31* | NM_138811 | 8 | c.C758T | p.S253F | 0 | 1.34 | -1.17 | 0 |
| JM696 | Missense | *NUDCD1* | NM_001128211 | 6 | c.A800G | p.D267G | 0 | 1.94 | -0.46 | 0 |
| JM889 | Missense | *PHF20L1* | NM_001277196 | 20 | c.G2971A | p.V991I | 1 | 5.75 | 1.78 | 0 |
| JM206 | Missense | *KIAA2026* | NM_001017969 | 3 | c.T1576C | p.C526R | NA | NA | NA | 0 |
| JM202 | Missense | *CNTLN* | NM_017738 | 24 | c.C4028G | p.T1343R | NA | NA | NA | 0 |
| JM213 | Missense | *DCAF12* | NM_015397 | 5 | c.G668C | p.R223T | 0 | 2.41 | 1.51 | 0 |
| JM144 | Missense | *KIF24* | NM_194313 | 11 | c.C3551G | p.S1184C | 0 | 1.43 | -1.57 | 0 |
| JM1357 | Missense | *NOTCH1* | NM_017617 | 8 | c.T1430A | p.I477N | 1 | 7.47 | 4.48 | 0 |
| JM155 | Missense | *PTPN11* | NM_001330437 | 3 | c.A182G | p.D61G | 1 | 4.99 | 3.43 | 0 |
| JM217 | Missense | *EDNRB* | NM_003991 | 7 | c.G1198A | p.G400R | 0.02 | 2.68 | 1.81 | 0 |
| JM172 | Missense | *EIF2AK4* | NM_001013703 | 4 | c.G458A | p.R153Q | 0 | 4.88 | 2.42 | 1.66E-05 |
| JM0002 | Missense | *EID1* | NM_014335 | 1 | c.G142T | p.G48W | 0.65 | 1.74 | 0.29 | 8.38E-06 |
| JM258 | Missense | *ABCA3* | NM_001089 | 28 | c.G4327A | p.G1443R | 0 | 4.06 | 0.93 | 5.79E-05 |
| JM0024 | Missense | *MBTPS1* | NM_003791 | 11 | c.G1342A | p.A448T | 0.13 | 4.73 | -2.04 | 1.76E-05 |
| JM1401 | Missense | *DBF4B* | NM_145663 | 14 | c.T1681A | p.L561M | 0 | 1.6 | -0.15 | 0 |
| JM141 | Missense | *SS18* | NM_001007559 | 5 | c.A569G | p.Y190C | 0.06 | 0.78 | 0.67 | 0 |
| JM141 | Missense | *FARSA* | NM_004461 | 6 | c.C616A | p.P206T | 0 | 2.93 | 1.58 | 0 |
| JM174 | Missense | *ZNF146* | NM_001099638 | 3 | c.T676C | p.S226P | 0.81 | 2.27 | 2.4 | 0 |
| JM174 | Missense | *SRC* | NM_005417 | 4 | c.G61A | p.A21T | 0 | 1.12 | 0.09 | 0 |
| JM187 | Missense | *CHRNA4* | NM_000744 | 5 | c.C721T | p.R241W | 0.02 | 2.21 | 1.9 | 0 |
| JM214 | Missense | *MCM3AP* | NM_003906 | 25 | c.C5372T | p.S1791L | 0.84 | 5.81 | -0.95 | 1.65E-05 |
| JM198 | Missense | *MN1* | NM_002430 | 1 | c.A675T | p.Q225H | 0.65 | 2.32 | 1.97 | 0 |
| JM140 | stoploss | *GDPD4* | NM_182833 | 16 | c.T1561A | p.X521K | 0 | 1.08 | -1.12 | 0 |
| JM0024 | Silent | *GPR174* | NM_032553 | 1 | c.C604T | p.L202L | 0.89 | 2.59 | 0.39 | 0 |
| JM892 | Silent | *SLC6A17* | NM_001010898 | 12 | c.C2067T | p.P689P | 0.96 | 3.84 | 2.74 | 0 |
| JM206 | Silent | *BROX* | NM_001288579 | 6 | c.A450G | p.A150A | 0 | 2.24 | -0.92 | 3.32E-05 |
| JM155 | Silent | *NVL* | NM_001243146 | 13 | c.A1230G | p.A410A | 0 | 2.73 | 0.87 | 0 |
| JM187 | Silent | *C3orf18* | NM_001171740 | 2 | c.C174T | p.G58G | 0.01 | 0.57 | -0.2 | 8.35E-06 |
| JM177 | Silent | *PLAG1* | NM_001114635 | 3 | c.G240A | p.T80T | 0.83 | 2.81 | 2.03 | 3.31E-05 |
| JM0043 | Silent | *TOR1B* | NM_001317893 | 3 | c.C591T | p.Y197Y | 0.74 | 2.56 | 1.11 | 1.65E-05 |
| JM185 | Silent | *FCN2* | NM_015837 | 4 | c.C282T | p.L94L | 0 | 0.23 | -0.72 | 0 |
| JM172 | Silent | *WDFY4* | NM_020945 | 19 | c.C3423T | p.S1141S | NA | NA | NA | 0 |
| JM199 | Silent | *CLMP* | NM_024769 | 7 | c.C1044T | p.V348V | 0 | 1.82 | 0.79 | 0 |
| JM1419 | Silent | *SCAF11* | NM_004719 | 11 | c.G966A | p.R322R | 1 | 5.68 | -1.7 | 0 |
| JM202 | Silent | *LIN7A* | NM_004664 | 2 | c.A90G | p.A30A | 0.04 | 2 | 0.54 | 0 |
| JM0051 | Silent | *MYO5C* | NM_018728 | 22 | c.G2721A | p.G907G | 0 | 3.9 | -0.42 | 0 |
| JM217 | Silent | *NOB1* | NM_014062 | 6 | c.C717T | p.F239F | 0 | 0.52 | -0.29 | 0 |
| JM1357 | Silent | *FA2H* | NM_024306 | 4 | c.A612G | p.T204T | 0.73 | 2.98 | 1.42 | 0 |
| JM206 | Silent | *ELL* | NM_006532 | 4 | c.C381T | p.Y127Y | 0 | 1.5 | -1.46 | 0 |
| JM740 | Silent | *CD22* | NM_001185101 | 9 | c.G1608A | p.K536K | 0.51 | 2.54 | 0.12 | 0 |
| JM213 | Silent | *PPP1R13L* | NM_001142502 | 5 | c.G741A | p.P247P | 0 | 2.77 | 2.38 | 8.27E-06 |
| JM177 | Silent | *ADAMTS1* | NM_006988 | 8 | c.T2196C | p.T732T | 0 | 4.55 | -0.02 | 2.48E-05 |
| JM169 | Silent | *PRMT2* | NM_001535 | 8 | c.G849A | p.E283E | 0.75 | 3.43 | 2.11 | 8.24E-06 |
